# A review on microRNA detection and expression studies in dogs

**DOI:** 10.3389/fvets.2023.1261085

**Published:** 2023-10-05

**Authors:** Mara S. Varvil, Andrea Pires dos Santos

**Affiliations:** ^1^Department of Comparative Pathobiology, Purdue University, West Lafayette, IN, United States; ^2^Department of Veterinary Clinical Sciences, Washington State University, Pullman, WA, United States

**Keywords:** microRNA, miRNA, canine, neoplasia, cancer, infectious diseases, inflammatory disease, developmental disease

## Abstract

MicroRNAs (miRNAs) are small non-coding RNAs that function by post-transcriptional regulation of gene expression. Their stability and abundance in tissue and body fluids makes them promising potential tools for both the diagnosis and prognosis of diseases and attractive therapeutic targets in humans and dogs. Studies of miRNA expression in normal and disease processes in dogs are scarce compared to studies published on miRNA expression in human disease. In this literature review, we identified 461 peer-reviewed papers from database searches using the terms “canine,” “dog,” “miRNA,” and “microRNA”; we screened 244 for inclusion criteria and then included a total of 148 original research peer-reviewed publications relating to specific miRNA expression in canine samples. We found an overlap of miRNA expression changes between the four groups evaluated (normal processes, non-infectious and non-inflammatory conditions, infectious and/or inflammatory conditions, and neoplasia) in 39 miRNAs, 83 miRNAs in three of the four groups, 110 miRNAs in two of the three groups, where 158 miRNAs have only been reported in one of the groups. Additionally, the mechanism of action of these overlapping miRNAs varies depending on the disease process, elucidating a need for characterization of the mechanism of action of each miRNA in each disease process being evaluated. Herein we also draw attention to the lack of standardization of miRNA evaluation, consistency within a single evaluation method, and the need for standardized methods for a direct comparison.

## Introduction

MicroRNAs (miRNAs), first discovered in 1993 (1), are short (18–24 nucleotide) non-coding RNAs that perform regulatory functions through post-transcriptional regulation of gene expression (2–4). While individual miRNAs are not required for individual tissue development, they are often required to maintain homeostasis (2). MiRNAs are essential modulators of cell differentiation, proliferation, and function, and their expression is often altered in disease states such as cancer, metabolic disease, and with response to infectious agents (2). Gene expression studies have demonstrated such alterations, and functional studies have also linked miRNA dysregulation as a factor in disease progression (2).

The numerous alterations of miRNA in disease provide great potential for using miRNAs as diagnostic biomarkers (2). MiRNAs are stable and can be recovered from formalin-fixed, paraffin-embedded (FFPE) sections and other sources where overall RNA quality may be low (2). MiRNAs are often released from cells in exosomes and microvesicles or circulate bonded to lipoproteins and RNA-protein complexes and thus are available in body fluids, including plasma, saliva, and urine (2, 5–21). MiRNAs have also been detected in feces, tears, breast milk, bronchial lavage fluid, colostrum, seminal, amniotic, pleural, peritoneal, and cerebrospinal fluids (22). These characteristics make miRNAs suitable for diagnostic and prognostic testing. Additionally, as miRNA signatures in disease processes become known, efforts are being made to directly target dysregulated miRNAs to treat disease with either miRNA mimics or inhibitors, depending on the dysregulated miRNA and therapeutic goal (2, 23).

Although there are more studies published on miRNA expression in human disease than the veterinary counterpart, animal models are often used to elucidate the roles of miRNAs in oncogenesis and progression (24), as such, many similarities are found in miRNA expression between human and dog diseases. MiRNA expression studies have been performed on normal and abnormal canine tissues to evaluate physiological processes occurring during normal development and disease processes (24).

In dogs, aberrant miRNA expression has been identified in many cancer types, including but not limited to, lymphoma, mammary cancers, mast cell tumor, urothelial carcinoma, osteosarcoma, melanoma, and leukemia (24). While individual studies report miRNA expression in a specific disease process, miRNAs are often involved in numerous regulatory processes and altered expression may result in upregulation in one disease and downregulation in another (2–4). To date, most studies of miRNAs in dogs have generally evaluated one miRNA or a small group of miRNAs associated with a particular disease state or developmental process. The goal of this review was to describe what is reported concerning miRNA in dogs across normal physiologic processes (NP), non-infectious and non-inflammatory disease processes (NDP), infectious and/or inflammatory disease processes (IDP), and neoplasia. Here we concisely describe miRNA and their expression in both physiologic processes and disease states.

## Materials and methods

### Data acquisition

A search was performed on PubMed and Google Scholar for the terms “canine” OR “dog” AND “miRNA” OR “microRNA.” Publications available in the English language were considered. Review articles were not included in this study; however, original research publications cited within review articles were evaluated for inclusion in this review. Publications were screened for potential applicability.

### Initial screening

A total of 461 reports were identified and further evaluated. Of the 461 reports identified, 224 were found to be not applicable (i.e., not evaluating dogs or not evaluating miRNAs) and 13 were unavailable for review. The remaining 224 were evaluated for inclusion eligibility. Criteria for inclusion in the review were 1. manuscript must be peer-reviewed, 2. manuscript must report evaluation of miRNA in canine samples, 3. manuscript must present original research (other literature review papers are not included in this literature review), and 4. manuscript must report expression evaluation relating to specific miRNA (i.e., blanket statements of miRNA being increased or decreased without discussion of individual miRNAs were not included in this review).

### Results

**Figure 1:**
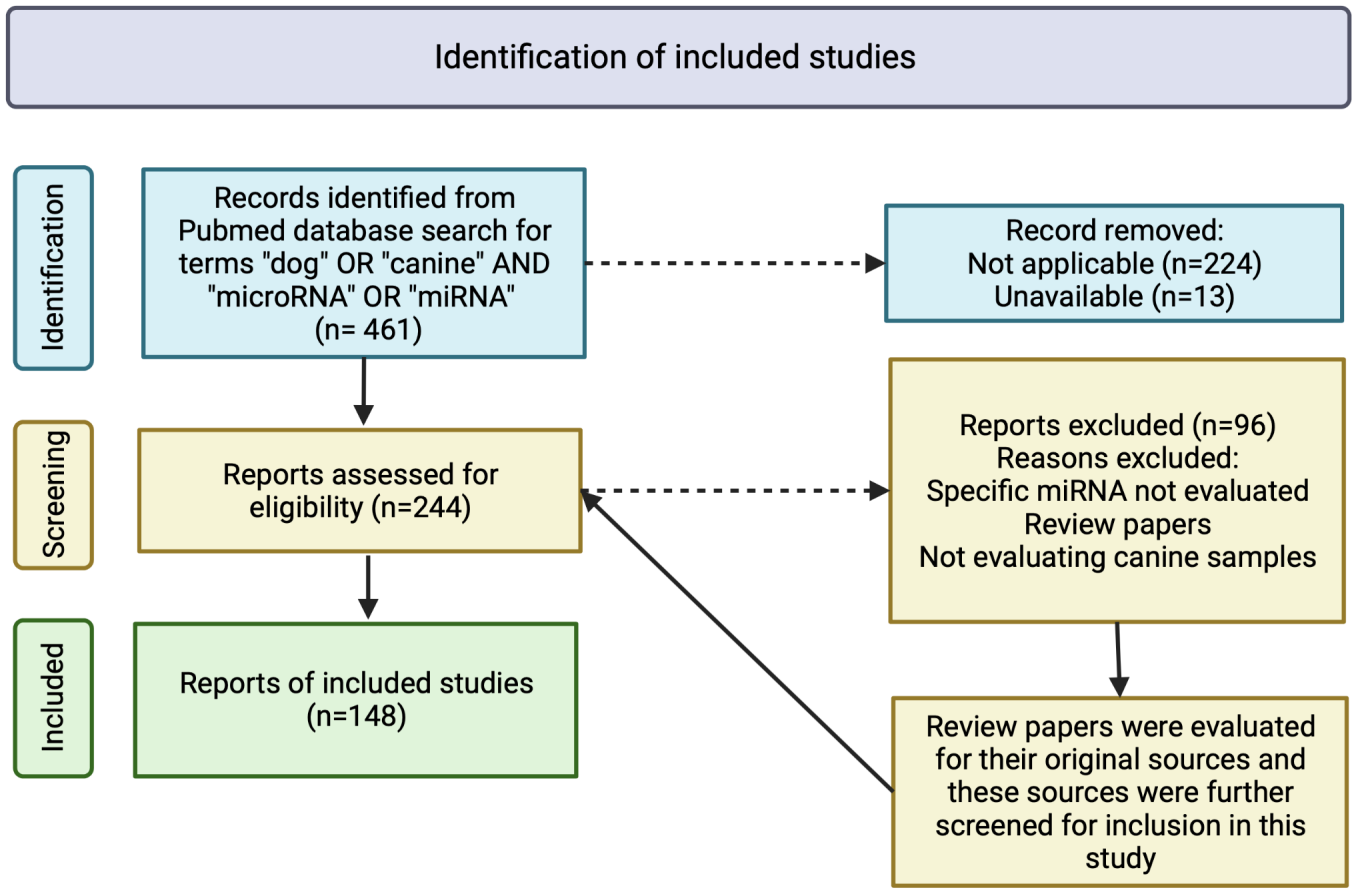
Flowchart of inclusion criteria and evaluation of peer-reviewed publications on detection and expression of microRNA in dogs. A total of 461 manuscripts were initially identified, with 237 excluded due to inapplicability or unavailability. Two-hundred and forty-four manuscripts were further assessed for eligibility with 96 being excluded due to inapplicability, not evaluating specific miRNA or the manuscripts being review papers. References of review papers were evaluated for applicability in this study. A total of 148 manuscripts were included in this review. Image created with biorender.com.

A total of 148 peer-reviewed publications were included in this study (Figure 1). MiRNA expression in neoplasia is the most frequently published, with a total of 63 publications, while miRNA expression in NDP is the second most published, with a total of 55 publications. MiRNA expression in IDP represents 27 publications (Table 1). MiRNA expression in NP is the least represented in this review, with 10 publications (Table 1).

**Table 1:** Stratification of miRNAs and publications within the four groups: normal processes, noninfectious/non-inflammatory disease processes, infectious/inflammatory disease processes (NP, NDP, IDP, respectively) and neoplasia and experimental evaluation of functions and miRNA targets.

	Number of miRNAs	Number of publications
NP	157	10
NDP	175	55
IDP	126	27
Neoplasia	325	63
Evaluation of function and targets	74	40

**Figure 2:**
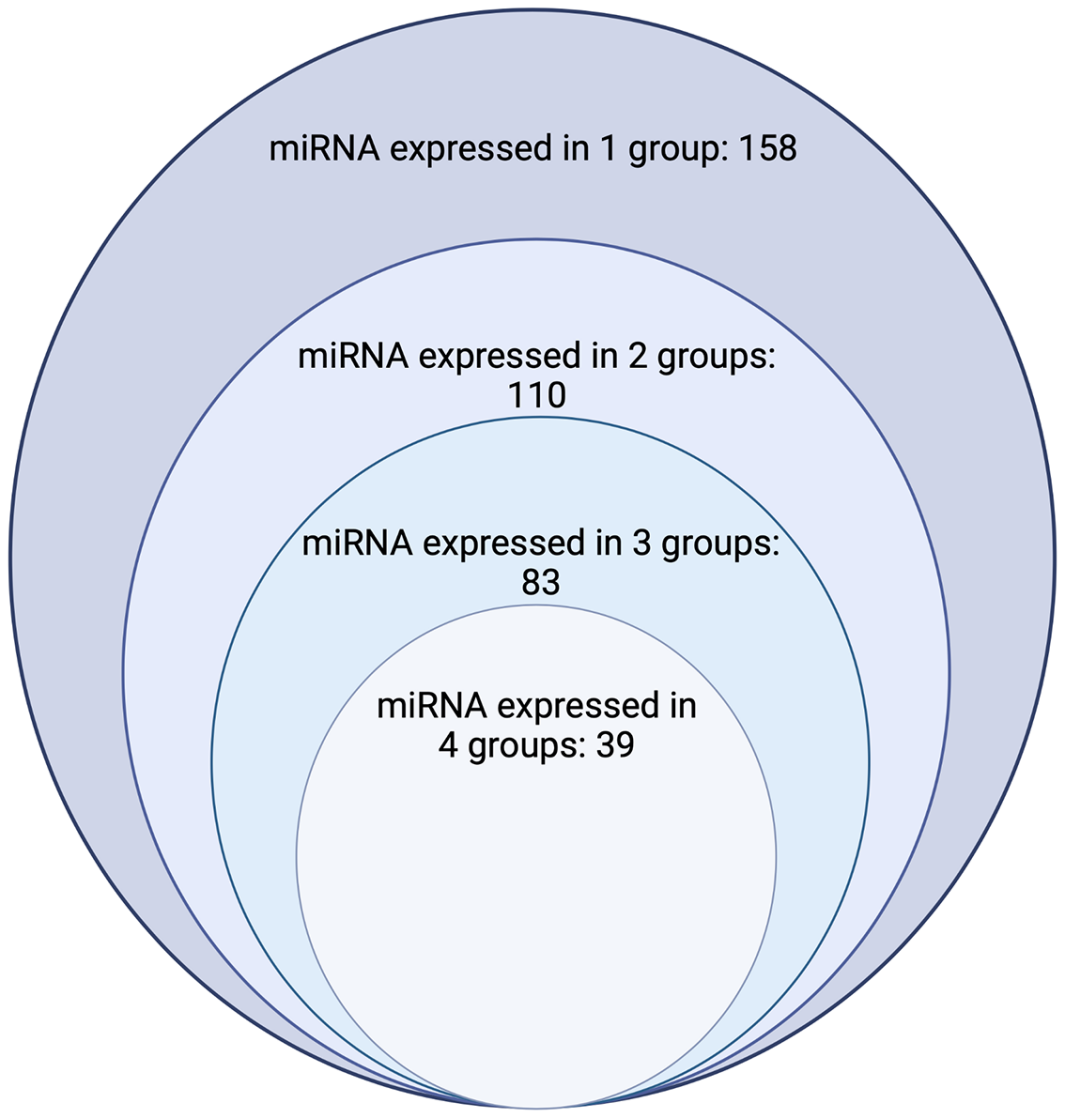
Chart depicting the number of dog miRNAs with expression documented in one, two, three, and four groups. The largest circle represents the number of miRNAs where expression has been found in only one group (*n* = 158), the second largest circle represents the number of miRNAs which have had expression characterized in two of the four groups (Normal processes, noninfectious/noninflammatory disease processes, infectious/inflammatory disease processes, and neoplasia) considered (*n* = 110). The third largest/s smallest circle represents the number of miRNAs which have had expression characterized in three of the four groups (*n* = 83). The smallest circle represents the number of miRNAs who have had expression characterized in all four groups considered (*n* = 39) Image created with biorender.com.

The publications included in this review reported the expression of a total of 391 miRNAs. Of those, 157 miRNAs are categorized in NP (Table 1; Supplementary Table S1), 175 miRNAs in NDP (Table 1; Supplementary Table S2), 126 miRNAs in IDP (Table 1; Supplementary Table S3), and 325 miRNAs in neoplasia (Table 1; Supplementary Table S4). Regarding overlapping, 158 miRNAs were studied in only one of the categories (NP, NDP, IDP, or neoplasia), 110 miRNAs were reported in two of the categories, 83 miRNAs reported in three, and 39 miRNAs were reported in all four groups (Table 1; Figure 2).

Forty articles included in this review also characterized the miRNA functions, targets, and/or affected pathways (Supplementary Table S5) of various miRNAs, including 14/39 of the miRNAs reported in all four of the groups.

MiR-1, expressed with both upregulation and downregulation in NDP (25–28) and neoplasia (29–36), expression in NP (37–39), and downregulation in IDP (40), targets both MET in hepatocellular carcinoma (33) and EDFR in mammary carcinoma (35, 41), and was shown to inhibit cell proliferation in a MET dependent manner (33). MiR-20a, reported to be expressed in NP (37, 42), upregulated in various neoplasms (7, 41, 43, 44), and downregulated in both NDP (45) and IDP (46), targets TGF-β affecting epithelial to mesenchymal transition and fibrosis in myxomatous mitral valve disease (45). MiR-214 was found to be both upregulated and downregulated in NP (37, 47) and neoplasia (7, 8, 41, 44, 48–52), downregulated in NDP (28), upregulated in IDP (40, 53), targets COP1, affecting the P53 pathways for apoptosis in hemangiosarcoma (50). Both miR-148a (enriched in NP (38), upregulated in NDP (54–56) and IDP (57), and both upregulated and downregulated in neoplasia (35, 41, 58–60)) and miR-205 [enriched in NP (38), downregulated in IDP (40), and both upregulated and downregulated in NDP (61–63) and neoplasia (24, 35, 41, 52, 60, 64–67)] target ERBB3 in mammary carcinoma and oral melanoma, respectively (41, 52, 58, 64, 65) while miR-205 has also been shown to target EGFR in mammary carcinoma (35, 41) and ECFC affecting NOTCH2 and promoting angiogenesis in distraction osteogenesis (61).

The majority of studies used only a single method of evaluation including reverse transcription-quantitative polymerase chain reaction (RT-qPCR) (8, 10–13, 16, 17, 19, 27, 31, 33, 36, 37, 40–43, 47, 49, 50, 52, 54, 55, 59, 60, 62, 68–121, 25–), microarray (44, 53, 122–125), or next generation sequencing (NGS) (32, 38, 126–131), Some studies evaluated the miRNA through two methods including NGS with validation *via* RT-qPCR (29, 30, 35, 39, 40, 45, 46, 51, 56, 61, 63, 65, 67, 132–144),microarray with validation *via* RT-qPCR (5, 9, 14, 18, 28, 57, 58, 64, 66, 145–149), one study utilized NGS with validation using digital drop PCR (ddPCR) (7), one study utilized nanostring with qRT-PCR (20), and two studies utilized *in situ* hybridization (ISH) for validation of their findings (39, 109).

## Discussion

Several miRNAs have been evaluated in canine tissues from cytologic samples, histologic, and fecal samples, and body fluids, including plasma and serum, as well as canine cell lines. As reported in this literature review, an overlap of miRNA expression has been found in various disease processes.

The overlap in expression profiles of miRNA in various disease processes and normal tissues elucidates the need for further characterization of the mechanisms the miRNA used to regulate each disease process and their function in normal tissues to better utilize them as diagnostic, therapeutic, and prognostic tools. A single miRNA may negatively regulate multiple target proteins through interaction with different target mRNAs (23). Defining the targets of miRNAs becomes vital for understanding the biological role of the miRNAs and for identifying potential uses for therapeutic and diagnostic agents (2).

In this review we did not consider the effects differences of geographic location on miRNA expression and how differening treatment protocols and disease prevalences within different global locations may affect miRNA expression, as this is beyond the scope of this review. However, it should be considered that the treatment and management method of individual disease processes as well as environmental factors, which often vary by geographical location, may alter that pathophysiologic behavior of any one process which then could be reflected by a different expression panel of miRNAs.

Another limitation of this review is the variety of methods for evaluating miRNAs in dogs. In addition to a lack of standardization within the methods for evaluating miRNA, there is also a lack of standardization in how the normalization of RT-qPCR is performed. The differing normalization methods may reflect differences in the outcome of miRNA expression findings (150). Additionally, several studies utilized RNU6 as a reference gene when normalizing their RT-qPCR data (5, 10, 16, 18, 25, 28, 33, 39, 46, 47, 60–63, 65, 66, 79, 80, 82, 84, 85, 87, 92, 97, 101, 107–109, 113, 114, 116, 120, 135, 139–141, 143, 147, 149), which, since RNU6 is not a miRNA and therefore may not behave as a miRNA, has been suggested to lead to inaccurate or skewed results (151, 152). Additionally, some studies chose to normalize their data to their exogenous reference gene or spike in control (19, 55, 142). Recently, it has been recommended to evaluate each tissue individually to find the best miRNA to use as a reference gene using programs like NormFinder or GeNORM (153). Additional normalization has also been reported using the mean of miRNAs expressed (154). The lack of standardization in evaluating of miRNA raises concerns for studies with results that are not repeatable by independent sources.

## Conclusion

This literature review characterizes the peer-reviewed literature on miRNA expression in dogs across four categories (NP, NDP, IDP, and neoplasia). Herein we have highlighted the overlap of miRNA expression in various disease processes tissues and that miRNA expression is dependent on disease process.

## Author contributions

MV: Conceptualization, Formal analysis, Investigation, Methodology, Project administration, Visualization, Writing – original draft, Writing – review & editing. AS: Conceptualization, Project administration, Supervision, Visualization, Writing – review & editing.
