# Supplementary material for: A review on microRNA detection and expression studies in dogs
Source: Front Vet Sci. 2023 Oct 5;10:1261085. doi: 10.3389/fvets.2023.1261085 (PMC10585042; doi:10.3389/fvets.2023.1261085)
Supplement: Supplementary file 1 [file Data_Sheet_1.zip › Table S1.DOCX]

**Table S1.** miRNA expressed in normal physiologic processes and normal tissues (NP) of dogs. Tissues reported include: Adrenal cortex of the pituitary gland, bone Marrow, brain, cerebrospinal fluid, colon, fecal material, ileum, jejunum, kidney, liver, pancreas, pituitary gland (no specification of region), plasma, retina, sciatic nerve, striated muscle, testicular tissue, and thymus.

| **miRNA** | **Expression/enrichment/detectability** | **Life stage/specific tissue section** |  |
| --- | --- | --- | --- |
| **Adrenal Cortex** (130) | | | |
| **miR-7** | High expression | Healthy adult dogs |  |
| **miR-26a** | High expression | Healthy adult dogs |  |
| **miR-26b** | High expression | Healthy adult dogs |  |
| **miR-30a** | High expression | Healthy adult dogs |  |
| **miR-30d** | High expression | Healthy adult dogs |  |
| **miR-99a** | High expression | Healthy adult dogs |  |
| **Bone Marrow** (38) | | | |
| **miR-7** | High tissue enrichment | Adult bone marrow tissue |  |
| **miR-129** | Tissue enrichment | Adult bone marrow tissue |  |
| **miR-144** | High tissue enrichment | Adult bone marrow tissue |  |
| **miR-374b** | Tissue enrichment | Adult bone marrow tissue |  |
| **miR-450a** | Tissue enrichment | Adult bone marrow tissue |  |
| **miR-450b** | Tissue enrichment | Adult bone marrow tissue |  |
| **miR-451** | Tissue enrichment | Adult bone marrow tissue |  |
| **miR-628** | Tissue enrichment | Adult bone marrow tissue |  |
| **miR-8865** | High tissue enrichment | Adult bone marrow tissue |  |
| **Brain** (38)^74^ | | | |
| **miR-7** | High tissue enrichment | Adult brain tissue |  |
| **miR-9** | High tissue enrichment | Adult brain tissue |  |
| **miR-9a** | High tissue enrichment | Adult brain tissue |  |
| **miR-92b** | High tissue enrichment | Adult brain tissue |  |
| **miR-105** | High tissue enrichment | Adult brain tissue |  |
| **miR-105a** | High tissue enrichment | Adult brain tissue |  |
| **miR-105b** | High tissue enrichment | Adult brain tissue |  |
| **miR-107** | Tissue enrichment | Adult brain tissue |  |
| **miR-124** | High tissue enrichment | Adult brain tissue |  |
| **miR-127** | Tissue enrichment | Adult brain tissue |  |
| **miR-128** | High tissue enrichment | Adult brain tissue |  |
| **miR-129** | High tissue enrichment | Adult brain tissue |  |
| **miR-132** | High tissue enrichment | Adult brain tissue |  |
| **miR-135a** | Tissue enrichment | Adult brain tissue |  |
| **miR-136** | High tissue enrichment | Adult brain tissue |  |
| **miR-137** | High tissue enrichment | Adult brain tissue |  |
| **miR-138a** | High tissue enrichment | Adult brain tissue |  |
| **miR-139** | High tissue enrichment | Adult brain tissue |  |
| **miR-146b** | Tissue enrichment | Adult brain tissue |  |
| **miR-149** | High tissue enrichment | Adult brain tissue |  |
| **miR-153** | Tissue enrichment | Adult brain tissue |  |
| **miR-184** | High tissue enrichment | Adult brain tissue |  |
| **miR-212** | High tissue enrichment | Adult brain tissue |  |
| **miR-219** | High tissue enrichment | Adult brain tissue |  |
| **miR-323** | High tissue enrichment | Adult brain tissue |  |
| **miR-331** | Tissue enrichment | Adult brain tissue |  |
| **miR-335** | Tissue enrichment | Adult brain tissue |  |
| **miR-338** | Tissue enrichment | Adult brain tissue |  |
| **miR-342** | Tissue enrichment | Adult brain tissue |  |
| **miR-380** | High tissue enrichment | Adult brain tissue |  |
| **miR-382** | Tissue enrichment | Adult brain tissue |  |
| **miR-383** | Tissue enrichment | Adult brain tissue |  |
| **miR-410** | High tissue enrichment | Adult brain tissue |  |
| **miR-411** | Tissue enrichment | Adult brain tissue |  |
| **miR-431** | High tissue enrichment | Adult brain tissue |  |
| **miR-432** | Tissue enrichment | Adult brain tissue |  |
| **miR-487a** | High tissue enrichment | Adult brain tissue |  |
| **miR-487b** | High tissue enrichment | Adult brain tissue |  |
| **miR-504** | Tissue enrichment | Adult brain tissue |  |
| **miR-628** | Tissue enrichment | Adult brain tissue |  |
| **miR-744** | Tissue enrichment | Adult brain tissue |  |
| **miR-873** | Tissue enrichment | Adult brain tissue |  |
| **miR-874** | High tissue enrichment | Adult brain tissue |  |
| **miR-885** | Tissue enrichment | Adult brain tissue |  |
| **miR-889** | High tissue enrichment | Adult brain tissue |  |
| **miR-1249** | Tissue enrichment | Adult brain tissue |  |
| **miR-1298** | High tissue enrichment | Adult brain tissue |  |
| **Cardiac Tissue** (38, 39) | | | |
| **Let-7c** | Enriched | Cardiac valve (39) |  |
| **miR-1** | Enriched | Myocardium (39) |  |
|  | Tissue enrichment | Adult cardiac tissue (38) |  |
| **miR-30e** | Enriched | Myocardium (39) |  |
| **miR-99b** | Enriched | Cardiac valve (39) |  |
| **miR-125b** | Enriched | Cardiac valve (39) |  |
| **miR-127** | Enriched | Cardiac valve (39) |  |
| **miR-133a** | Enriched | Myocardium (39) |  |
|  | High tissue enrichment | Adult cardiac tissue (38) |  |
| **miR-133b** | Tissue enrichment | Adult cardiac tissue (39) |  |
| **miR-199a** | Enriched | Cardiac valve (39) |  |
| **miR-204** | Enriched | Cardiac valve (39) |  |
| **miR-208b** | Enriched | Myocardium (39) |  |
|  | High tissue enrichment | Adult cardiac tissue (38) |  |
| **miR-320** | Enriched | Cardiac valve (39) |  |
| **miR-328** | Enriched | Cardiac valve (39) |  |
| **miR-499** | Enriched | Myocardium (39) |  |
|  | High tissue enrichment | Adult cardiac tissue (38) |  |
| **miR-744** | Enriched | Cardiac valve (39) |  |
| **Cerebrospinal fluid** (13) | | | |
| **miR-19b** | Detectable | Healthy adult dogs |  |
| **miR-103** | Detectable | Healthy adult dogs |  |
| **miR-127** | Detectable | Healthy adult dogs |  |
| **Colon** (38) | | | |
| **miR-147** | Tissue enrichment | Adult tissue |  |
| **Fecal material** (42) | | | |
| **miR-16** | Detectable | Healthy dogs |  |
| **miR-20a** | Detectable | Healthy dogs |  |
| **miR-92a** | Detectable | Healthy dogs |  |
| **Ileum** (38) | | | |
| **miR-15a** | Tissue enrichment | Adult tissue |  |
| **miR-147** | Tissue enrichment | Adult tissue |  |
| **miR-215** | Tissue enrichment | Adult tissue |  |
| **Jejunum** (38) | | | |
| **miR-141** | Tissue enrichment | Adult tissue |  |
| **miR-215** | Tissue enrichment | Adult tissue |  |
| **miR-342** | Tissue enrichment | Adult tissue |  |
| **miR-374b** | Tissue enrichment | Adult tissue |  |
| **miR-802** | Tissue enrichment | Adult tissue |  |
| **Kidney** (129) | | | |
| **Let-7f** | Highly expressed | Renal cortex |  |
| **miR-9** | Highly expressed | Renal medulla |  |
| **miR-10a** | Highly expressed | Renal medulla |  |
| **miR-27a** | Highly expressed | Renal medulla |  |
| **miR-34a** | Highly expressed | Renal medulla |  |
| **miR-129** | Highly expressed | Renal medulla |  |
| **miR-132** | Highly expressed | Renal medulla |  |
| **miR-135a** | Highly expressed | Renal medulla |  |
| **miR-152** | Highly expressed | Renal medulla |  |
| **miR-181b** | Highly expressed | Renal medulla |  |
| **miR-193b** | Highly expressed | Renal cortex |  |
| **miR-194** | Highly expressed | Renal cortex |  |
| **miR-196a** | Highly expressed | Renal medulla |  |
| **miR-203** | Highly expressed | Renal medulla |  |
| **miR-204** | Highly expressed | Renal cortex |  |
| **miR-212** | Highly expressed | Renal medulla |  |
| **miR-300** | Highly expressed | Renal medulla |  |
| **miR-323** | Highly expressed | Renal cortex |  |
| **miR-377** | Highly expressed | Renal cortex |  |
| **miR-378** | Highly expressed | Renal cortex |  |
| **miR-379** | Highly expressed | Renal cortex |  |
| **miR-382** | Highly expressed | Renal cortex |  |
| **miR-425** | Highly expressed | Renal cortex |  |
| **miR-486** | Highly expressed | Renal medulla |  |
| **miR-490** | Highly expressed | Renal cortex and renal medulla |  |
| **miR-676** | Highly expressed | Renal cortex |  |
| **miR-889** | Highly expressed | Renal cortex |  |
| **Liver** (38, 155) | | | |
| **miR-122** | High tissue enrichment | Adult tissue (38) |  |
| **miR-127** | Detectable | Healthy adult dogs (155) |  |
| **miR-148a** | Tissue enrichment | Adult hepatic tissue (38) |  |
| **miR-433** | Detectable | Healthy adult dogs (155) |  |
| **miR-885** | Tissue enrichment | Adult tissue (38) |  |
| **miR-3591** | High tissue enrichment | Adult tissue (38) |  |
| **Pancreas** (38) | | | |
| **miR-135a** | Tissue enrichment | Adult pancreatic tissue |  |
| **miR-141** | Tissue enrichment | Adult pancreatic tissue |  |
| **miR-148a** | Tissue enrichment | Adult pancreatic tissue |  |
| **miR-153** | Tissue enrichment | Adult pancreatic tissue |  |
| **miR-216a** | High tissue enrichment | Adult pancreatic tissue |  |
| **miR-216b** | High tissue enrichment | Adult pancreatic tissue |  |
| **miR-217** | High tissue enrichment | Adult pancreatic tissue |  |
| **miR-375** | High tissue enrichment | Adult pancreatic tissue |  |
| **miR-802** | Tissue enrichment | Adult pancreatic tissue |  |
| **Pituitary Gland** (130) | | | |
| **miR-7** | High expression | Healthy adult dogs |  |
| **miR-30c** | High expression | Healthy adult dogs |  |
| **miR-30d** | High expression | Healthy adult dogs |  |
| **miR-124** | High expression | Healthy adult dogs |  |
| **miR-125b** | High expression | Healthy adult dogs |  |
| **Plasma** (38) | | | |
| **miR-15a** | Tissue enrichment | Adult plasma |  |
| **miR-16** | High tissue enrichment | Adult plasma |  |
| **miR-25** | Tissue enrichment | Adult plasma |  |
| **miR-107** | Tissue enrichment | Adult plasma |  |
| **miR-144** | High tissue enrichment | Adult plasma |  |
| **miR-331** | Tissue enrichment | Adult plasma |  |
| **miR-383** | Tissue enrichment | Adult plasma |  |
| **miR-423** | High tissue enrichment | Adult plasma |  |
| **miR-451** | Tissue enrichment | Adult plasma |  |
| **Retina** (146) | | | |
| **miR-29b** | Upregulated | Normal aged retina |  |
| **Sciatic nerve** (38) | | | |
| **miR-127** | Tissue enrichment | Adult sciatic nerve |  |
| **miR-184** | High tissue enrichment | Adult sciatic nerve |  |
| **miR-193a** | Tissue enrichment | Adult sciatic nerve |  |
| **miR-338** | Tissue enrichment | Adult sciatic nerve |  |
| **miR-382** | Tissue enrichment | Adult sciatic nerve |  |
| **miR-411** | Tissue enrichment | Adult Sciatic nerve t |  |
| **miR-432** | Tissue enrichment | Adult sciatic nerve |  |
| **miR-504** | Tissue enrichment | Adult sciatic nerve t |  |
| **miR-744** | Tissue enrichment | Adult sciatic nerve |  |
| **miR-1249** | Tissue enrichment | Adult sciatic nerve |  |
| **Striated muscle** (38) | | | |
| **miR-1** | Tissue enrichment | Adult tissue |  |
| **miR-133a** | High tissue enrichment | Adult tissue |  |
| **miR-133b** | Tissue enrichment | Adult tissue |  |
| **miR-206** | High tissue enrichment | Adult tissue |  |
| **miR-208b** | High tissue enrichment | Adult tissue |  |
| **Testicular tissue** (37, 38, 47) | | | |
| **Let-7a** | Upregulated | Retinoic acid dependent spermatogenesis compared to non-retinoic acid dependent spermatogenesis (47) |  |
| **Let-7b** | Upregulated | Retinoic acid dependent spermatogenesis compared to non-retinoic acid dependent spermatogenesis (47) |  |
| **Let-7c** | Upregulated | Retinoic acid dependent spermatogenesis compared to non-retinoic acid dependent spermatogenesis (47) |  |
| **Let-7f** | Upregulated | Retinoic acid dependent spermatogenesis compared to non-retinoic acid dependent spermatogenesis (47) |  |
| **Let-7g** | Upregulated | Retinoic acid dependent spermatogenesis compared to non-retinoic acid dependent spermatogenesis (47) |  |
| **miR-1** | Upregulated | Adult testicular tissue compared to immature testicular tissue (37) |  |
| **miR-7** | Upregulated | Adult testicular tissue compared to immature testicular tissue (37) |  |
| **miR-9** | Upregulated | Adult testicular tissue compared to immature testicular tissue (37) |  |
| **miR-15a** | Upregulated | Adult testicular tissue compared to immature testicular tissue (37) |  |
| **miR-15b** | Upregulated | Adult testicular tissue compared to immature testicular tissue (37) |  |
| **miR-16** | Upregulated | Adult testicular tissue compared to immature testicular tissue (37) |  |
| **miR-18a** | Upregulated | Adult testicular tissue compared to immature testicular tissue (37) |  |
| **miR-19a** | Upregulated | Adult testicular tissue compared to immature testicular tissue (37) |  |
|  | Downregulated | Retinoic acid dependent spermatogenesis compared to non-retinoic acid dependent spermatogenesis (47) |  |
| **miR-20a** | Upregulated | Adult testicular tissue compared to immature testicular tissue (37) |  |
| **miR-22** | Upregulated | Adult testicular tissue compared to immature testicular tissue (37) |  |
| **miR-23a** | Upregulated | Retinoic acid dependent spermatogenesis compared to non-retinoic acid dependent spermatogenesis (47) |  |
| **miR-23b** | Upregulated | Retinoic acid dependent spermatogenesis compared to non-retinoic acid dependent spermatogenesis (47) |  |
| **miR-29b** | Upregulated | Adult testicular tissue compared to immature testicular tissue (37) |  |
| **miR-29b** | Downregulated | Retinoic acid dependent spermatogenesis compared to non-retinoic acid dependent spermatogenesis (47) |  |
| **miR-29c** | Upregulated | Adult testicular tissue compared to immature testicular tissue (37) |  |
|  | Downregulated | Retinoic acid dependent spermatogenesis compared to non-retinoic acid dependent spermatogenesis (47) |  |
| **miR-34a** | Upregulated | Retinoic acid dependent spermatogenesis compared to non-retinoic acid dependent spermatogenesis (47) |  |
| **miR-34b** | Upregulated | Adult testicular tissue compared to immature testicular tissue (37) |  |
|  | Upregulated | Retinoic acid dependent spermatogenesis compared to non-retinoic acid dependent spermatogenesis (47) |  |
|  | High tissue enrichment | Adult tissue (38) |  |
| **miR-34c** | Upregulated | Adult testicular tissue compared to immature testicular tissue (37) |  |
|  | Upregulated | Retinoic acid dependent spermatogenesis compared to non-retinoic acid dependent spermatogenesis (47) |  |
|  | High tissue enrichment | Adult tissue (38) |  |
| **miR-96** | Upregulated | Adult testicular tissue compared to immature testicular tissue (37) |  |
| **miR-101** | Upregulated | Adult testicular tissue compared to immature testicular tissue (37) |  |
|  | Downregulated | Retinoic acid dependent spermatogenesis compared to non-retinoic acid dependent spermatogenesis (47) |  |
| **miR-106** | Upregulated | Adult testicular tissue compared to immature testicular tissue (37) |  |
| **miR-106a** | Tissue enrichment | Adult tissue (38) |  |
| **miR-122** | Upregulated | Adult testicular tissue compared to immature testicular tissue (37) |  |
| **miR-124** | Upregulated | Adult testicular tissue compared to immature testicular tissue (37) |  |
| **miR-125a** | Upregulated | Retinoic acid dependent spermatogenesis compared to non-retinoic acid dependent spermatogenesis (47) |  |
| **miR-130a** | Downregulated | Adult testicular tissue compared to immature testicular tissue (37) |  |
| **miR-133b** | Upregulated | Adult testicular tissue compared to immature testicular tissue (37) |  |
| **miR-136** | Downregulated | Adult testicular tissue compared to immature testicular tissue (37) |  |
| **miR-137** | Downregulated | Retinoic acid dependent spermatogenesis compared to non-retinoic acid dependent spermatogenesis (47) |  |
| **miR-140** | Upregulated | Adult testicular tissue compared to immature testicular tissue (37) |  |
| **miR-141** | Upregulated | Retinoic acid dependent spermatogenesis compared to non-retinoic acid dependent spermatogenesis (47) |  |
| **miR-143** | Upregulated | Adult testicular tissue compared to immature testicular tissue (37) |  |
| **miR-145** | Upregulated | Adult testicular tissue compared to immature testicular tissue (37) |  |
| **miR-146a** | Upregulated | Retinoic acid dependent spermatogenesis compared to non-retinoic acid dependent spermatogenesis (47) |  |
| **miR-146b** | Upregulated | Adult testicular tissue compared to immature testicular tissue (37) |  |
|  | Upregulated | Retinoic acid dependent spermatogenesis compared to non-retinoic acid dependent spermatogenesis (37) |  |
| **miR-146b** | Tissue enrichment | Adult tissue (38) |  |
| **miR-148b** | Downregulated | Adult testicular tissue compared to immature testicular tissue (37) |  |
| **miR-181** | Downregulated | Adult testicular tissue compared to immature testicular tissue (37) |  |
| **miR-181b** | Downregulated | Adult testicular tissue compared to immature testicular tissue (37) |  |
| **miR-183** | Downregulated | Adult testicular tissue compared to immature testicular tissue (37) |  |
| **miR-184** | Upregulated | Retinoic acid dependent spermatogenesis compared to non-retinoic acid dependent spermatogenesis (47) |  |
| **miR-190b** | Upregulated | Adult testicular tissue compared to immature testicular tissue (37) |  |
| **miR-192** | Upregulated | Adult testicular tissue compared to immature testicular tissue (37) |  |
| **miR-200** | Upregulated | Retinoic acid dependent spermatogenesis compared to non-retinoic acid dependent spermatogenesis (47) |  |
| **miR-200a** | Upregulated | Adult testicular tissue compared to immature testicular tissue (37) |  |
| **miR-200a** | Upregulated | Retinoic acid dependent spermatogenesis compared to non-retinoic acid dependent spermatogenesis (37) |  |
| **miR-200c** | Upregulated | Retinoic acid dependent spermatogenesis compared to non-retinoic acid dependent spermatogenesis (37) |  |
|  | Upregulated | Retinoic acid dependent spermatogenesis compared to non-retinoic acid dependent spermatogenesis (37) |  |
| **miR-202** | High tissue enrichment | Adult tissue (38) |  |
| **miR-203** | Downregulated | Adult testicular tissue compared to immature testicular tissue (37) |  |
| **miR-205** | High tissue enrichment | Adult tissue (38) |  |
| **miR-210** | Upregulated | Adult testicular tissue compared to immature testicular tissue (37) |  |
| **miR-214** | Downregulated | Adult testicular tissue compared to immature testicular tissue (37) |  |
|  | Upregulated | Retinoic acid dependent spermatogenesis compared to non-retinoic acid dependent spermatogenesis (47) |  |
| **miR-335** | Upregulated | Adult testicular tissue compared to immature testicular tissue (37) |  |
|  | Tissue enrichment | Adult tissue (38) |  |
| **miR-375** | Upregulated | Adult testicular tissue compared to immature testicular tissue (37) |  |
| **miR-377** | Upregulated | Adult testicular tissue compared to immature testicular tissue (37) |  |
| **miR-449a** | High tissue enrichment | Adult tissue (38) |  |
| **miR-449b** | High tissue enrichment | Adult tissue (38) |  |
| **miR-450a** | Tissue enrichment | Adult tissue (38) |  |
| **miR-450b** | Tissue enrichment | Adult tissue (38) |  |
| **miR-506** | High tissue enrichment | Adult tissue (38) |  |
| **miR-507b** | High tissue enrichment | Adult tissue (38) |  |
| **miR-508a** | High tissue enrichment | Adult tissue (38) |  |
| **miR-508b** | High tissue enrichment | Adult tissue (38) |  |
| **miR-873** | Tissue enrichment | Adult tissue (38) |  |
| **miR-8831** | High tissue enrichment | Adult tissue (38) |  |
| **miR-8908a** | High tissue enrichment | Adult tissue (38) |  |
| **miR-8908b** | High tissue enrichment | Adult tissue (38) |  |
| **miR-8908c** | High tissue enrichment | Adult tissue (38) |  |
| **Thymus** (38) | | | |
| **miR-106a** | Tissue enrichment | Adult thymic tissue |  |
| **miR-193a** | Tissue enrichment | Adult thymic tissue |  |
| **miR-205** | High tissue enrichment | Adult thymic tissue |  |
