# Supplementary material for: A review on microRNA detection and expression studies in dogs
Source: Front Vet Sci. 2023 Oct 5;10:1261085. doi: 10.3389/fvets.2023.1261085 (PMC10585042; doi:10.3389/fvets.2023.1261085)
Supplement: Supplementary file 1 [file Data_Sheet_1.zip › Table S2.DOCX]

**Table S2.** MiRNA expressed in noninfectious and noninflammatory disease processes (NDP) of dogs. Abbreviations: CNS: Central nervous system; CSF: Cerebral spinal fluid; ERD: Early retinal degeneration; PRCD: Progressive rod-cone degeneration; RCD: Rod cone dysplasia; XLHN: X-linked hereditary nephropathy; Xlpra2: X-linked progressive retinal atrophy.

| **Disease process** | **Sample types** | **miRNA** | **Regulation** |
| --- | --- | --- | --- |
| **Bone conditions** | | | |
| Bone regeneration in bone defects | Blood-derived mesenchymal stem cells | **miR-21** | Upregulated (118) |
| **Cardiac Conditions** | | | |
| Atrial fibrillation | Atrial tissue | **Let-7** | Downregulated (148) |
|  |  | **miR-15** | Downregulated (148) |
|  |  | **miR-21** | Upregulated (148) |
|  |  | **miR-29b** | Downregulated (77) |
|  |  | **miR-23** | Upregulated (148) |
|  |  | **miR-199** | Upregulated (148) |
|  |  | **mR-328** | Upregulated (156) |
|  | Atrial cells | **miR-30** | Downregulated (95, 148) |
|  |  | **miR-133** | Downregulated (95) |
|  | Cardiac fibroblasts (atrial) | **miR-132** | Downregulated (106) |
|  | Epicardial fat pad | **miR-7** | Downregulated (63) |
|  |  | **miR-21** | Upregulated (63) |
|  |  | **miR-34c** | Downregulated (63) |
|  |  | **miR-124a** | Downregulated (63) |
|  |  | **miR-129** | Downregulated (63) |
|  |  | **miR-137** | Downregulated (63) |
|  |  | **miR-138a** | Downregulated (63) |
|  |  | **miR-200c** | Downregulated (63) |
|  |  | **miR-203** | Downregulated (63) |
|  |  | **miR-205** | Downregulated (63) |
|  |  | **miR-206** | Upregulated (63) |
|  |  | **miR-208b** | Upregulated (63) |
|  |  | **miR-224** | Upregulated (63) |
|  |  | **miR-432** | Downregulated (63) |
|  |  | **miR-450b** | Upregulated (63) |
|  | Myocardial cells | **miR-205** | Upregulated (62) |
| Mild to moderate cardiac enlargement (11) | Serum | **Let-7b** | Upregulated |
|  |  | **Let-7c** | Upregulated |
|  |  | **miR-103** | Upregulated |
|  |  | **miR-301b** | Downregulated |
|  |  | **miR-329a** | Downregulated |
|  |  | **miR-379** | Downregulated |
|  |  | **miR-486** | Downregulated |
|  |  | **miR-487** | Downregulated |
|  |  | **miR-574** | Downregulated |
|  |  | **miR-802** | Downregulated |
| Concentric cardiac hypertrophy (108) | Serum | **Let-7b** | Upregulated |
|  |  | **miR-375** | Upregulated |
| Congestive heart failure | Serum (11) | **Let-7b** | Upregulated |
|  |  | **Let-7c** | Upregulated |
|  |  | **miR-103** | Upregulated |
|  |  | **miR-329a** | Downregulated |
|  |  | **miR-379** | Downregulated |
|  |  | **miR-486** | Downregulated |
|  |  | **miR-487** | Downregulated |
|  |  | **miR-486** | Downregulated |
|  |  | **miR-487** | Downregulated |
|  |  | **miR-574** | Downregulated |
|  |  | **miR-802** | Downregulated |
|  | Plasma (138) | **miR-133** | Upregulated |
|  | Atrial fibroblasts (105) | **miR-26a** | Downregulated |
|  | Left ventricular cardiomyocytes (25) | **miR-30a** | Upregulated |
|  |  | **miR-21** | Upregulated |
|  |  | **miR-146b** | Upregulated |
|  | Left ventricular fibroblasts (25) | **Let-7e** | Upregulated |
|  |  | **miR-1** | Upregulated |
|  |  | **miR-21** | Upregulated |
|  |  | **miR-30a** | Upregulated |
|  |  | **miR-30c** | Downregulated |
|  |  | **miR-124** | Upregulated |
|  |  | **miR-125a** | Upregulated |
|  |  | **miR-128** | Downregulated |
|  |  | **miR-133a** | Upregulated |
|  |  | **miR-133b** | Upregulated |
|  |  | **miR-141** | Downregulated |
|  |  | **miR-146b** | Upregulated |
|  |  | **miR-208b** | Upregulated |
|  |  | **miR-421** | Downregulated |
| Degenerative valvular disease stage C (90) | Plasma | **miR-30b** | Downregulated |
|  |  | **miR-125** | Downregulated |
|  |  | **miR-126** | Downregulated |
|  |  | **miR-133b** | Downregulated |
| Dilated cardiomyopathy (18) | Serum | **Let-7c** | Downregulated |
|  |  | **Let-7f** | Upregulated |
|  |  | **miR-21** | Upregulated |
|  |  | **miR-30c** | Downregulated |
|  |  | **miR-101** | Upregulated |
|  |  | **miR-188** | Downregulated |
| Doxorubicin induced cardiotoxicity (126) | Plasma | **miR-107** | Downregulated |
|  |  | **miR-146a** | Downregulated |
|  |  | **miR-181a** | Upregulated |
|  |  | **miR-502** | Upregulated |
| Ischemia and reperfusion injury in cardiopulmonary bypass patients (28) | Left ventricular myocardium | **Let-7e** | Downregulated |
|  |  | **miR-1** | Downregulated |
|  |  | **miR-23a** | Downregulated |
|  |  | **miR-23b** | Downregulated |
|  |  | **miR-24** | Downregulated |
|  |  | **miR-214** | Downregulated |
|  |  | **miR-451** | Downregulated |
|  |  | **miR-489** | Downregulated |
|  |  | **miR-499** | Downregulated |
|  |  | **miR-574** | Downregulated |
| Left bundle branch block (96) | Left ventricular wall | **miR-133a** | Downregulated |
| Myxomatous mitral valve disease | Serum | **Let-7b** | Upregulated (11) |
|  |  | **Let-7b** | Downregulated (125) |
|  |  | **Let-7c** | Upregulated (11) |
|  |  | **Let-7g** | Downregulated (125) |
|  |  | **miR-19b** | Downregulated (125) |
|  |  | **miR-30c** | Downregulated (125) |
|  |  | **miR-30d** | Downregulated (125) |
|  |  | **miR-103** | Upregulated (11) |
|  |  | **miR-130b** | Upregulated (108, 125) |
|  |  | **miR-151** | Downregulated (125) |
|  |  | **miR-301b** | Downregulated (132) |
|  |  | **miR-329a** | Downregulated (11) |
|  |  | **miR-375** | Downregulated (125) |
|  |  | **miR-379** | Downregulated (11) |
|  |  | **miR-425** | Downregulated (125) |
|  |  | **miR-486** | Downregulated (11) |
|  |  | **miR-487** | Downregulated (11) |
|  |  | **miR-486** | Downregulated (11) |
|  |  | **miR-487** | Downregulated (11) |
|  |  | **miR-574** | Downregulated (11) |
|  |  | **miR-802** | Downregulated (11) |
|  | Plasma | **miR-9** | Upregulated (21) |
|  |  | **miR-30b** | Upregulated (69) |
|  |  | **miR-181a** | Upregulated (21) |
|  |  | **miR-495** | Upregulated (21) |
|  |  | **miR-599** | Downregulated (21) |
|  | Mitral valve | **Let-7c** | Downregulated (45) |
|  |  | **miR-17** | Downregulated (45) |
|  |  | **miR-20a** | Downregulated (45) |
|  |  | **miR-30d** | Downregulated (45 |
| Nicotine-induced atrial remodeling (111) | Atrial cells | **miR-132** | Downregulated |
|  |  | **miR-582** | Downregulated |
| Patent ductus arteriosus, pulmonic stenosis (108) | Serum | **miR-130b** | Upregulated |
| Right ventricular heart failure (124) | Ventricular cardiomyocytes | **miR-21** | Upregulated |
|  |  | **miR-221** | Upregulated |
| **Endocrine conditions** (17) | | | |
| Pituitary dependent Hypercortisolism | Plasma | **miR-122** | Upregulated |
|  |  | **miR-141** | Upregulated |
|  |  | **miR-222** | Upregulated |
|  |  | **miR-375** | Upregulated |
|  |  | **miR-483** | Upregulated |
| Adrenal-dependent hypercortisolism | Plasma | **miR-223** | Downregulated |
|  |  | **miR-483** | Upregulated |
| **Hepatobiliary conditions** | | | |
| Biliary mucocele (6) | Serum | **miR-21** | Upregulated |
|  |  | **miR-122** | Upregulated |
| Hepatic fibrosis (80) | Serum | **miR-34a** | Upregulated |
|  |  | **miR-122** | Upregulated |
| Hepatic steatosis (80) | Serum | **miR-34a** | Upregulated |
|  |  | **miR-122** | Upregulated |
| Liver disease (not otherwise specified) (103) | Serum | **miR-122** | Upregulated |
| Liver injury (55) | Serum | **miR-122** | Upregulated |
|  |  | **miR-148a** | Upregulated |
| Portosystemic shunt (81) | Serum | **miR-21** | Upregulated |
|  |  | **miR-34a** | Upregulated |
|  |  | **miR-122** | Upregulated |
|  |  | **miR-126** | Upregulated |
| **Musculoskeletal conditions** | | | |
| Canine X-linked muscular dystrophy (26) | Serum | **miR-1** | Upregulated |
|  |  | **miR-133a** | Upregulated |
|  |  | **miR-206** | Upregulated |
| Distraction osteogenesis (61) | Bony callus tissue | **miR-205** | Downregulated |
| Duchenne muscular dystrophy (112) | Serum, Tibialis cranialis muscle, diaphragm | **miR-187** | Upregulated |
|  | Serum | **miR-499** | Upregulated |
| Golden Retriever muscular dystrophy (9) | Serum | **miR-136** | Upregulated |
|  |  | **miR-377** | Upregulated |
|  |  | **miR-381** | Upregulated |
|  |  | **miR-410** | Upregulated |
|  |  | **miR-411** | Upregulated |
|  |  | **miR-412** | Upregulated |
|  |  | **miR-431** | Upregulated |
|  |  | **miR-433** | Upregulated |
|  |  | **miR-487** | Upregulated |
|  |  | **miR-495** | Upregulated |
|  |  | **miR-543** | Upregulated |
|  |  | **miR-655** | Upregulated |
|  |  | **miR-656** | Upregulated |
|  |  | **miR-889** | Upregulated |
|  | Biceps femoris | **miR-222** | Upregulated |
|  |  | **miR-486** | Downregulated |
| **Neurologic conditions** | | | |
| Cervical spondylomyelopathy (20) | CSF | **miR-224** | Upregulated |
|  |  | **miR-302d** | Downregulated |
|  |  | **miR-494** | Downregulated |
|  |  | **miR-592** | Downregulated |
|  |  | **miR-758** | Upregulated |
|  |  | **miR-4454** | Downregulated |
|  |  | **miR-4455** | Downregulated |
|  |  | **miR-6529** | Downregulated |
| CNS disease (not otherwise specified) (13) | CSF | **miR-10b** | Upregulated |
|  |  | **miR-19b** | Detected |
|  |  | **miR-103** | Detected |
|  |  | **miR-124** | Detected |
|  |  | **miR-127** | Detected |
| Degenerative myelopathy | Plasma (14) | **miR-26b** | Upregulated |
|  | Spinal cord (27) | **Let-7e** | Downregulated |
|  |  | **miR-1** | Downregulated |
|  |  | **miR-23a** | Upregulated |
|  |  | **miR-95** | Downregulated |
|  |  | **miR-105b** | Downregulated |
|  |  | **miR-133** | Downregulated |
|  |  | **miR-142** | Upregulated |
|  |  | **miR-149** | Downregulated |
|  |  | **miR-181b** | Downregulated |
|  |  | **miR-206** | Downregulated |
|  |  | **miR-216a** | Downregulated |
|  |  | **miR-219** | Downregulated |
|  |  | **miR-221** | Upregulated |
|  |  | **miR-324** | Downregulated |
|  |  | **miR-325** | Downregulated |
|  |  | **miR-326** | Downregulated |
|  |  | **miR-329a** | Downregulated |
|  |  | **miR-494** | Downregulated |
|  |  | **miR-539** | Downregulated |
|  |  | **miR-874** | Downregulated |
|  |  | **miR-1842** | Downregulated |
| Induced ischemic brain injury (54) | Serum | **miR-1** | Downregulated |
|  |  | **miR-132** | Downregulated |
|  |  | **miR-300** | Downregulated |
|  |  | **miR-371** | Downregulated |
|  |  | **miR-384** | Downregulated |
|  |  | **miR-424** | Downregulated |
|  |  | **miR-450a** | Upregulated |
|  |  | **miR-490** | Downregulated |
|  |  | **miR-628** | Upregulated |
|  |  | **miR-758** | Downregulated |
|  | Urine | **miR-27b** | Upregulated |
|  |  | **miR-138b** | Upregulated |
|  |  | **miR-148a** | Upregulated |
|  |  | **miR-363** | Upregulated |
|  |  | **miR-487b** | Upregulated |
|  |  | **miR-491** | Downregulated |
|  |  | **miR-499** | Upregulated |
|  |  | **miR-874** | Downregulated |
| **Nutritional conditions** | | | |
| Calorie restricted diet in overweight dogs (89) | Adipose tissue | **miR-21** | Upregulated |
|  |  | **miR-103** | Downregulated |
|  |  | **miR-107** | Downregulated |
|  | Muscle biopsy | **miR-23** | Downregulated |
|  |  | **miR-107** | Downregulated |
| Supplementation with commercial hepatoprotectant (87) | Serum | **miR-122** | Downregulated |
| **Ocular conditions** (146) | | | |
| Age-related macular degeneration | Retina | **miR-7** | Upregulated |
|  |  | **miR-155** | Upregulated |
| ERD | Retina | **miR-19a** | Upregulated |
|  |  | **miR-20** | Upregulated |
|  |  | **miR-29b** | Upregulated |
| RCD1 | Retina | **miR-19a** | Upregulated |
|  |  | **miR-20** | Upregulated |
| Xlpra2 | Retina | **Let-7** | Upregulated |
|  |  | **miR-7** | Upregulated |
|  |  | **miR-15a** | Upregulated |
|  |  | **miR-16** | Upregulated |
|  |  | **miR-19a** | Upregulated |
|  |  | **miR-20** | Upregulated |
|  |  | **miR-34b** | Downregulated |
|  |  | **miR-146b** | Upregulated |
|  |  | **miR-147** | Upregulated |
|  |  | **miR-208b** | Downregulated |
| Mutant retinas (Xlpra2, RCD1, ERD, PRCD) | Retina | **miR-7** | Upregulated |
|  |  | **miR-21** | Upregulated |
|  |  | **miR-155** | Upregulated |
| **Pancreatic conditions** | | | |
| Exocrine pancreatic injury (56) | Serum | **miR-148a** | Upregulated |
|  |  | **miR-375** | Upregulated |
| Acute pancreatic injury (94) | Serum | **miR-215** | Upregulated |
|  |  | **miR-375** | Upregulated |
| **Renal conditions** | | | |
| Kidney disease (Not otherwise specified) (128) | Urinary exosomes | **miR-10b** | Upregulated |
|  |  | **miR-21a** | Upregulated |
|  |  | **miR-486a** | Upregulated |
|  |  | **miR-3107** | Upregulated |
| XLHN (134) | Kidney | **miR-21** (75) | Upregulated |
|  |  | **miR-142** | Upregulated |
|  |  | **miR-146b** | Upregulated |
|  |  | **miR-147** | Upregulated |
|  |  | **miR-802** | Upregulated |
| XLHN at onset of azotemia (134) | Kidney | **miR-96** | Upregulated |
|  |  | **miR-146a** | Upregulated |
|  |  | **miR-182** | Upregulated |
|  |  | **miR-183** | Upregulated |
|  |  | **miR-215** | Downregulated |
|  |  | **miR-335** | Upregulated |
|  |  | **miR-370** | Upregulated |
|  |  | **miR-380** | Upregulated |
|  |  | **miR-381** | Upregulated |
|  |  | **miR-410** | Upregulated |
|  |  | **miR-451** | Upregulated |
|  |  | **miR-889** | Upregulated |
| XLHN at onset of proteinuria (134) | Kidney | **miR-19a** | Upregulated |
|  |  | **miR-101** | Upregulated |
|  |  | **miR-105b** | Downregulated |
|  |  | **miR-340** | Upregulated |
|  |  | **miR-590** | Upregulated |
|  |  | **miR-1301** | Downregulated |
|  |  | **miR-8890** | Downregulated |
| XLHN at onset of proteinuria and at onset of azotemia (134) | Kidney | **miR-29a** | Upregulated |
|  |  | **miR-31** | Upregulated |
|  |  | **miR-150** | Upregulated |
|  |  | **miR-184** | Downregulated |
|  |  | **miR-196a** | Downregulated |
| XLHN at onset of azotemia and after development of advanced chronic kidney disease (134) | Kidney | **miR-155** | Upregulated |
| XLHN after development of advanced chronic kidney disease (134) | Kidney | **miR-18a** | Upregulated |
|  |  | **miR-34a** | Upregulated |
|  |  | **miR-486** | Downregulated |
|  |  | **miR-708** | Upregulated |
| Gentamicin-induced acute kidney injury (19) | Urine | **miR-16** | Downregulated |
|  |  | **miR-30c** | Upregulated |
| **Reproductive conditions** | | | |
| Testicular toxicity induced by ethylene glycol monomethyl ether (143) | Serum | **miR-98** | Downregulated |
|  |  | **miR-134** | Upregulated |
|  |  | **miR-135a** | Downregulated |
|  |  | **miR-146b** | Upregulated |
|  |  | **miR-149** | Upregulated |
|  |  | **miR-151** | Downregulated |
|  |  | **miR-184** | Downregulated |
|  |  | **miR-216b** | Upregulated |
|  |  | **miR-331** | Downregulated |
|  |  | **miR-362** | Downregulated |
|  |  | **miR-370** | Upregulated |
|  |  | **miR-374b** | Downregulated |
|  |  | **miR-383** | Downregulated |
|  |  | **miR-409** | Upregulated |
|  |  | **miR-411** | Upregulated |
|  |  | **miR-455** | Upregulated |
|  |  | **miR-486** | Downregulated |
|  |  | **miR-499** | Downregulated |
|  |  | **miR-507b** | Upregulated |
|  |  | **miR-551a** | Upregulated |
| Benign Prostatic Hyperplasia (115) | PMBCs | **miR-129** | Downregulated |
| **Respiratory conditions** | | | |
| Obstructive sleep apnea canine model (157) | Primary pulmonary fibroblasts | **miR-185** | Downregulated |
| Pulmonic stenosis | Serum | **Let-7b** (125) | Downregulated |
|  |  | **miR-30c** (125) | Downregulated |
|  |  | **miR-130b** (108) | Upregulated |
|  |  | **miR-346** (125) | Upregulated |
|  |  | **miR-505** (125) | Downregulated |
| Pulmonary embolism (121) | Plasma | **miR-28** | Upregulated |
| **Stress related conditions** | | | |
| Exposure to search and rescue related stress (135) | Serum | **Let-7a** | Upregulated |
|  |  | **Let-7f** | Upregulated |
| **Vascular conditions** (79) | | | |
| Stenosis after stent retriever thrombectomy | Vessel | **miR-21** | Upregulated |
| Stenosis after stent retriever thrombectomy | Vessel | **miR-26a** | Upregulated |
| Stenosis after stent retriever thrombectomy | Vessel | **miR-29** | Upregulated |
