# Supplementary material for: A review on microRNA detection and expression studies in dogs
Source: Front Vet Sci. 2023 Oct 5;10:1261085. doi: 10.3389/fvets.2023.1261085 (PMC10585042; doi:10.3389/fvets.2023.1261085)
Supplement: Supplementary file 1 [file Data_Sheet_1.zip › Table S3.DOCX]

**Table S3**. miRNA expression in infectious and/or inflammatory disease processes (IDP) in dogs. Abbreviations: H5N1: highly pathogenic avian influenza A; H3N2: influenza A variant virus; IBD: inflammatory bowel disease; MDCK: Madin-Darby Canine Kidney cells; PBMCs: peripheral blood mononuclear cells.

| **Disease process** | **Sample types** | **miRNA** | **Regulation** |
| --- | --- | --- | --- |
| **Infectious conditions** | | | |
| Canine influenza virus | Alveolar macrophages (150) | **miR-125b** | Downregulated |
|  |  | **miR-151** | Downregulated |
|  | Bronchiolar epithelial cells (150) | **miR-125b** | Downregulated |
|  |  | **miR-151** | Downregulated |
|  | MDCK cells (141) | **miR-151** | Downregulated |
|  | Lung (144) | **Let-7** | Upregulated |
|  |  | **miR-143** | Upregulated |
|  | Trachea (144) | **Let-7** | Upregulated |
|  |  | **miR-143** | Upregulated |
| Distemper post treatment (76) | Whole blood | **miR-155** | Upregulated |
| Dirofilaria immitis (73) | Serum | **miR-34** | Upregulated |
|  |  | **miR-34c** | Upregulated |
| H5N1 (145) | Lung | **miR-133b** | Upregulated |
|  |  | **miR-143** | Upregulated |
|  |  | **miR-1306** | Downregulated |
|  |  | **miR-1343** | Downregulated |
|  |  | **miR-4742** | Upregulated |
|  |  | **miR-6721** | Upregulated |
|  |  | **miR-6902** | Upregulated |
| H3N2 (145) | Lung | **miR-133b** | Upregulated |
|  |  | **miR-1306** | Downregulated |
|  |  | **miR-1343** | Downregulated |
|  |  | **miR-4742** | Upregulated |
|  |  | **miR-6721** | Upregulated |
|  |  | **miR-6902** | Upregulated |
| Influenza B infection (141) | MDCK Cells | **miR-197** | Upregulated |
|  |  | **miR-215** | Upregulated |
|  |  | **miR-361** | Upregulated |
|  |  | **miR-1841** | Upregulated |
|  |  | **miR-1842** | Upregulated |
| Leishmaniasis | PBMCs | **miR-**23 (5) | Upregulated (Visceral form) |
|  |  | **miR-150** (5) | Downregulated (Visceral form) |
|  |  | **miR-210** (5) | Upregulated (*Leishmania infantum*) |
|  |  | **miR-192** (53) | Upregulate (Visceral form) |
|  |  | **miR-194** (5) | Upregulated (Visceral form) |
|  |  | **miR-188** (53) | Upregulated (*Leishmania infantum*) |
|  |  | **miR-206** (53) | Upregulated (*Leishmania infantum*) |
|  |  | **miR-214** (53) | Upregulated (*Leishmania infantum*) |
|  |  | **miR-302d** (53) | Upregulated (*Leishmania infantum*) |
|  |  | **miR-345** (53) | Upregulated (*Leishmania infantum*) |
|  |  | **miR-371** (5)^10^ | Upregulated (Visceral form) |
|  |  | **miR-424** (5) | Upregulated (Visceral form) |
|  |  | **miR-432** (53) | Upregulated (*Leishmania infantum*) |
|  |  | **miR-433** (53) | Upregulated (*Leishmania infantum*) |
|  |  | **miR-451** (5) | Upregulated (Visceral form) |
|  |  | **mir-489** (53) | Downregulated (*Leishmania infantum*) |
|  |  | **miR-493** (53) | Upregulated (*Leishmania infantum*) |
|  |  | **miR-503** (5) | Upregulated (Visceral form) |
|  |  | **miR-503** (53) | Downregulated (*Leishmania infantum*) |
|  |  | **miR-514** (53) | Upregulated (*Leishmania infantum*) |
|  |  | **miR-539** (53) | Upregulated (*Leishmania infantum*) |
|  |  | **miR-574** (5) | Downregulated (Visceral form) |
|  |  | **miR-1835** (53) | Upregulated (*Leishmania infantum*) |
|  | Plasma | **miR-346** (74) | Upregulated (*Leishmania infantum and Leishmania braziliensis*) |
|  | Canine macrophage like cells | **miR-346** (74) | Upregulated (*Leishmania infantum and Leishmania braziliensis*) |
|  | Serum exosomes | **miR-122** (12) | Downregulated |
|  | Splenic leukocytes | **miR-7** (57) | Upregulated |
|  |  | **miR-21** (57, 72) | Upregulated |
|  |  | **miR-125b** (57) | Downregulated |
|  |  | **miR-148a** (72) | Upregulated |
|  |  | **miR-150** (72) | Downregulated |
|  |  | **miR-612** (72) | Upregulated |
| Toxocara canis infection (40) | Liver | **Let-7g** | Downregulated |
|  |  | **miR-1** | Downregulated |
|  |  | **miR-7** | Downregulated |
|  |  | **miR-10a** | Downregulated |
|  |  | **miR-10b** | Upregulated |
|  |  | **miR-15b** | Downregulated |
|  |  | **miR-21** | Downregulated |
|  |  | **miR-23a** | Upregulated |
|  |  | **miR-23b** | Upregulated |
|  |  | **miR-25** | Downregulated |
|  |  | **miR-26b** | Downregulated |
|  |  | **miR-30a** | Downregulated |
|  |  | **miR-103** | Downregulated |
|  |  | **miR-125a** | Upregulated |
|  |  | **miR-127** | Downregulated |
|  |  | **miR-129** | Downregulated |
|  |  | **miR-133c** | Downregulated |
|  |  | **miR-135a** | Upregulated |
|  |  | **miR-136** | Downregulated |
|  |  | **miR-144** | Upregulated |
|  |  | **miR-145** | Upregulated |
|  |  | **miR-146a** | Downregulated/Upregulated |
|  |  | **miR-150** | Upregulated |
|  |  | **miR-151** | Upregulated |
|  |  | **miR-180** | Upregulated |
|  |  | **miR-194** | Upregulated |
|  |  | **miR-205** | Downregulated |
|  |  | **miR-206** | Downregulated |
|  |  | **miR-214** | Upregulated |
|  |  | **miR-223** | Downregulated |
|  |  | **miR-294** | Upregulated |
|  |  | **miR-318** | Upregulated |
|  |  | **miR-335** | Downregulated |
|  |  | **miR-336** | Upregulated |
|  |  | **miR-337** | Upregulated |
|  |  | **miR-342** | Upregulated |
|  |  | **miR-370** | Downregulated |
|  |  | **miR-371** | Upregulated |
|  |  | **miR-375** | Downregulated |
|  |  | **miR-379** | Downregulated |
|  |  | **miR-381** | Up/Downregulated |
|  |  | **miR-382** | Downregulated |
|  |  | **miR-411** | Downregulated |
|  |  | **miR-425** | Downregulated |
|  |  | **miR-433** | Downregulated |
|  |  | **miR-487b** | Downregulated |
|  |  | **miR-495** | Downregulated |
|  |  | **miR-497** | Upregulated |
|  |  | **miR-503** | Upregulated |
|  |  | **miR-676** | Downregulated |
|  |  | **miR-802** | Upregulated |
|  |  | **miR-885** | Downregulated |
|  |  | **miR-889** | Downregulated |
|  |  | **miR-1839** | Downregulated |
|  |  | **miR-8884** | Downregulated |
| **Noninfectious inflammatory conditions** | | | |
| Atopic Dermatitis | Plasma (70) | **miR-203** | Upregulated |
|  |  | **miR-483** | Upregulated |
|  | Skin biopsy (142) | **miR-106b** | Downregulated |
|  |  | **miR-155** | Downregulated |
|  |  | **miR-186** | Downregulated |
|  |  | **miR-192** | Upregulated |
|  |  | **miR-200a** | Downregulated |
|  |  | **miR-203** | Downregulated |
|  |  | **miR-215** | Upregulated |
|  |  | **miR-223** | Downregulated |
|  |  | **miR-383** | Upregulated |
|  |  | **miR-423** | Upregulated |
|  |  | **miR-429** | Downregulated |
| Hepatitis | Serum | **miR-21** (6) | Upregulated (Chronic) |
|  |  | **miR-29a** (110) | Upregulated (Chronic) |
|  |  | **miR-122** (110, 158) | Upregulated (Acute and chronic) |
| Inflammatory skin disease (97) | Epidermis | **miR-21** | Upregulated |
|  |  | **miR-142** | Upregulated |
|  |  | **miR-146a** | Upregulated |
|  |  | **miR-155** | Upregulated |
| Large intestinal IBD (92) | Colonic mucosa | **miR-16** | Upregulated |
|  |  | **miR-122** | Upregulated |
|  |  | **miR-145** | Downregulated |
|  |  | **miR-146b** | Upregulated |
|  |  | **miR-185** | Downregulated |
|  |  | **miR-191** | Downregulated |
|  |  | **miR-223** | Downregulated |
|  | Serum | **miR-145** | Downregulated |
|  |  | **miR-191** | Downregulated |
|  |  | **miR-223** | Downregulated |
| Meningoencephalomyelitis of unknown origin (85) | CSF | **miR-181b** | Upregulated |
| Otitis externa (139) | Cerumen | **miR-125b** | Downregulated |
|  |  | **miR-146a** | Downregulated |
|  |  | **miR-320a** | Downregulated |
|  |  | **miR-342** | Downregulated |
|  |  | **miR-375** | Downregulated |
|  |  | **miR-378a** | Downregulated |
| Peri-implantitis (46) | Gingival tissue | **Let-7c** | Downregulated |
|  |  | **Let-7e** | Upregulated |
|  |  | **Let-7g**^154,155^ | Downregulated |
|  |  | **miR-7** | Upregulated |
|  |  | **miR-9** | Downregulated |
|  |  | **miR-16** | Downregulated |
|  |  | **miR-20a** | Downregulated |
|  |  | **miR-23a** | Downregulated |
|  |  | **miR-26a** | Downregulated |
|  |  | **miR-27a**^154,155^ | Downregulated |
|  |  | **miR-27b** | Downregulated |
|  |  | **miR-29a** | Downregulated |
|  |  | **miR-92b** | Downregulated |
|  |  | **miR-93** | Downregulated |
|  |  | **miR-98** | Upregulated |
|  |  | **miR-101** | Downregulated |
|  |  | **miR-125a** | Downregulated |
|  |  | **miR-127** | Downregulated |
|  |  | **miR-142** | Upregulated |
|  |  | **miR-145** | Upregulated |
|  |  | **miR-145** | Downregulated |
|  |  | **miR-146a** | Downregulated |
|  |  | **miR-152** | Downregulated |
|  |  | **miR-200a** | Downregulated |
|  |  | **miR-204** | Downregulated |
|  |  | **miR-211** | Downregulated |
|  |  | **miR-340** | Downregulated |
|  |  | **miR-342** | Downregulated |
|  |  | **miR-361** | Downregulated |
|  |  | **miR-374a** | Downregulated |
|  |  | **miR-375** | Upregulated |
|  |  | **miR-429** | Downregulated |
|  |  | **miR-451** | Downregulated |
|  |  | **miR-452** | Upregulated |
|  |  | **miR-486** | Downregulated |
|  |  | **miR-500** | Upregulated |
|  |  | **miR-532** | Downregulated |
|  |  | **miR-1271** | Downregulated |
| Prostatitis (60) | Prostatic tissue | **miR-146a** | Upregulated |
