# Supplementary material for: A review on microRNA detection and expression studies in dogs
Source: Front Vet Sci. 2023 Oct 5;10:1261085. doi: 10.3389/fvets.2023.1261085 (PMC10585042; doi:10.3389/fvets.2023.1261085)
Supplement: Supplementary file 1 [file Data_Sheet_1.zip › Table S4.DOCX]

**Table S4.** MiRNA expression in neoplasia in dogs. Abbreviations: DLBCL: diffuse large B-cell lymphoma; FFPE: Formalin-fixed, paraffin-embedded tissue; FFT: fresh frozen tissue; LUTD: lower urinary tract disease; MDCK: Madin-Darby canine kidney cell; PBMCs: peripheral blood mononuclear cells; UC: urothelial carcinoma

| **Disease process** | **Sample** | **miRNA** | **Regulation** |
| --- | --- | --- | --- |
| General tumor marker (8) | Plasma | **miR-126** | Upregulated |
|  |  | **miR-214** | Upregulated (Most specific for sarcomas) |
| Angiosarcoma (49) | Angiosarcoma cell line | **miR-126** | Upregulated |
|  |  | **miR-214** | Upregulated |
| Brain tumors (100) | Brain tissue | **miR-190a** | Downregulated |
| CNS neoplastic disease (13) | CSF | **miR-10b** | Upregulated |
| Epithelial to mesenchymal transition (36) | MDCK cells | **miR-1** | Upregulated |
|  |  | **miR-181b** | Upregulated |
|  |  | **miR-193b** | Downregulated |
|  |  | **miR-199** | Downregulated |
|  |  | **miR-664** | Downregulated |
|  |  | **miR-765** | Downregulated |
|  |  | **miR-874** | Upregulated |
| Hypoxia in high-grade canine glioma (32) | Glioma cells | **miR-1** | Upregulated |
|  |  | **miR-133c** | Upregulated |
|  |  | **miR-210** | Upregulated |
|  |  | **miR-216a** | Upregulated |
|  |  | **miR-320** | Upregulated |
|  |  | **miR-494** | Upregulated |
| Glioma (15) | Plasma | **miR-15b** | Upregulated |
|  | Plasma extracellular vesicles | **miR-15b** | Upregulated |
|  |  | **miR-340** | Upregulated |
| Hemangiosarcoma | Hemangiosarcoma cell line | **miR-126** (49) | Upregulated |
|  |  | **miR-214** (49) | Upregulated |
|  |  | **miR-214** (50) | Downregulated |
|  | Splenic tissue | **miR-22** 127) | Upregulated |
|  |  | **miR-26a** (127) | Downregulated |
|  |  | **miR-30e** (127) | Upregulated |
|  |  | **miR-125b** (127) | Upregulated |
|  |  | **miR-139** (127) | Downregulated |
|  |  | **miR-149** (127) | Downregulated |
|  |  | **miR-202** (127) | Downregulated |
|  |  | **miR-214** (50) | Downregulated |
|  |  | **miR-363** (127) | Upregulated |
|  |  | **miR-451** (127) | Upregulated |
|  |  | **miR-502** (127) | Upregulated |
|  |  | **miR-527b** (127) | Upregulated |
|  |  | **miR-713** (127) | Upregulated |
|  |  | **miR-33b**  (127) | Upregulated |
|  |  | **miR-504** (127) | Upregulated |
| Hepatocellular carcinoma | Liver (33) | **Let-7a** | Downregulated |
|  |  | **Let-7g** | Downregulated |
|  |  | **miR-1** | Downregulated |
|  |  | **miR-10b** | Upregulated |
|  |  | **miR-21** | Upregulated |
|  |  | **miR-107** | Downregulated |
|  | Serum (6) | **miR-21** | Upregulated |
| Hepatic premalignant alterations triggered by human nephrotoxic aristolochic acid I (147) | Liver | **Let-7a** | Downregulated |
|  |  | **Let-7b** | Downregulated |
|  |  | **miR-24** | Downregulated |
|  |  | **miR-27a** | Upregulated |
| Histiocytic sarcoma (disseminated) (71) | Whole blood | **Let-7g** | Downregulated |
| DLBCL | Lymph node (FFPE) (82) | **Let-7a** | Downregulated |
|  |  | **Let-7b** | Downregulated |
|  |  | **Let-7c** | Downregulated |
|  |  | **Let-7e** | Downregulated |
|  |  | **Let-7f** | Downregulated |
|  |  | **Let-7g** | Downregulated |
|  |  | **miR-34a** | Upregulated |
|  |  | **miR-155** | Downregulated |
|  | Serum (43) | **miR-20a** | Upregulated |
|  |  | **miR-94** | Upregulated (progressive disease) |
| B-cell lymphoma | Lymph node (FFPE) (98) | **miR-17** | Upregulated |
|  | Lymph node (FFT) (98) | **miR-17** | Upregulated |
|  | Lymph node aspirate (59) | **miR-18a** | Downregulated |
|  |  | **miR-19a** | Downregulated |
|  |  | **miR-19b** | Downregulated |
|  |  | **miR-23** | Upregulated |
|  |  | **miR-26b** | Downregulated |
|  |  | **miR-30b** | Downregulated |
|  |  | **miR-34a** | Downregulated |
|  |  | **miR-99a** | Upregulated |
|  |  | **miR-125a** | Upregulated |
|  |  | **miR-125b** | Upregulated |
|  |  | **miR-130b** | Downregulated |
|  |  | **miR-145** | Upregulated |
|  |  | **miR-146a** | Upregulated |
|  |  | **miR-148a** | Upregulated |
|  |  | **miR-150** | Upregulated |
|  |  | **miR-181b** | Upregulated |
|  |  | **miR-423** | Downregulated |
|  | Lymphoid/leukemia cell line (44) | **miR-19a** | Upregulated |
|  |  | **miR-19b** | Upregulated |
|  |  | **miR-181a** | Downregulated |
|  |  | **miR-203** | Downregulated |
|  |  | **miR-204** | Downregulated |
|  |  | **miR-218** | Upregulated |
|  | Plasma (59) | **miR-23** | Upregulated |
|  |  | **miR-26b** | Downregulated |
|  |  | **miR-29a** | Downregulated |
|  |  | **miR-29b** | Downregulated |
|  |  | **miR-29c** | Downregulated |
|  |  | **miR-31** | Downregulated |
|  |  | **miR-34a** | Downregulated |
|  |  | **miR-99a** | Upregulated |
|  |  | **miR-125a** | Upregulated |
|  |  | **miR-125b** | Upregulated |
|  |  | **miR-143** | Upregulated |
|  |  | **miR-145** | Upregulated |
|  |  | **miR-182** | Downregulated |
|  |  | **miR-181d** | Upregulated |
|  |  | **miR-423a** | Upregulated |
| Lymphoma (not otherwise specified) | Lymphoid/leukemia cell line (44) | **miR-9** | Upregulated |
|  |  | **miR-10b** | Upregulated |
|  |  | **miR-16** | Upregulated |
|  |  | **miR-18a** | Upregulated |
|  |  | **miR-20** | Upregulated |
|  |  | **miR-92a** | Upregulated |
|  |  | **miR-101** | Upregulated |
|  |  | **miR-103a** | Upregulated |
|  |  | **miR-105a** | Upregulated |
|  |  | **miR-106a** | Upregulated |
|  |  | **miR-106b** | Upregulated |
|  |  | **miR-107** | Upregulated |
|  |  | **miR-125a** | Downregulated |
|  |  | **miR-135b** | Downregulated |
|  |  | **miR-145** | Downregulated |
|  |  | **miR-149** | Downregulated |
|  |  | **miR-150** | Downregulated |
|  |  | **miR-197** | Downregulated |
|  |  | **miR-199** | Downregulated |
|  |  | **miR-203** | Downregulated |
|  |  | **miR-214** | Downregulated |
|  |  | **miR-371** | Downregulated |
|  | Serum (84) | **Let-7b** | Downregulated |
|  |  | **miR-223** | Downregulated |
|  |  | **miR-25** | Downregulated |
|  | Spleen (68) | **miR-17** | Upregulated |
|  |  | **miR-155** | Upregulated |
|  | Lymphoma/leukemia exosomes (132) | **miR-350** | Upregulated |
| T-cell lymphoma | Lymphoma/leukemia cell line (44) | **miR-17** | Upregulated |
|  |  | **miR-20a** | Upregulated |
|  |  | **miR-105a** | Upregulated |
|  |  | **miR-149** | Downregulated |
|  |  | **miR-183** | Upregulated |
|  |  | **miR-186** | Downregulated |
|  |  | **miR-218** | Downregulated |
|  | Lymph node aspirate (59) | **miR-21** | Upregulated |
|  |  | **miR-26b** | Downregulated |
|  |  | **miR-99a** | Upregulated |
|  |  | **miR-125b** | Upregulated |
|  |  | **miR-130b** | Downregulated |
|  |  | **miR-150** | Upregulated |
|  |  | **miR-155** | Upregulated |
|  |  | **miR-181c** | Downregulated |
|  |  | **miR-182** | Downregulated |
|  | Lymph node (FFPE and FFT) (98) | **miR-181a** | Upregulated |
|  | Plasma (59) | **miR-23** | Upregulated |
|  |  | **miR-26b** | Downregulated |
|  |  | **miR-423a** | Upregulated |
|  | Small intestine (FFPE) (122) | **miR-18b** | Upregulated |
|  |  | **miR-19a** | Downregulated |
|  |  | **miR-20b** | Upregulated |
|  |  | **miR-34a** | Downregulated |
|  |  | **miR-130b** | Downregulated |
|  |  | **miR-141** | Downregulated |
|  |  | **miR-142** | Upregulated |
|  |  | **miR-192** | Downregulated |
|  |  | **miR-194** | Downregulated |
|  |  | **miR-203** | Downregulated |
|  |  | **miR-363** | Upregulated |
| Hepatocellular lymphoma | Serum (6) | **miR-21** | Upregulated |
| Multicentric lymphoma with liver involvement | Serum (159) | **miR-122** | Upregulated |
| B-cell chronic lymphocytic leukemia | Whole blood (86) | **miR-125b** | Upregulated |
| Lymphoma/leukemia (132) | Lymphoma/leukemia cells | **miR-151** | Upregulated |
|  | Lymphoma/leukemia exosomes | **miR-486** | Upregulated |
|  |  | **miR-8884** | Upregulated |
| Adenoma (30) | Mammary tissue | **miR-338** | Downregulated |
| Benign mixed tumor (30) | Mammary tissue | **miR-1** | Downregulated |
|  |  | **miR-133a** | Downregulated |
|  |  | **miR-133b** | Downregulated |
|  |  | **miR-133c** | Upregulated |
|  |  | **miR-208b** | Downregulated |
|  |  | **miR-216b** | Downregulated |
| Benign mammary tumors (58) | Mammary tissue | **miR-203** | Downregulated |
| Mammary gland tumors (29) | Mammary gland | **Let-7c** | Downregulated |
|  |  | **miR-1** | Downregulated |
|  |  | **miR-9** | Downregulated |
|  |  | **miR-23b** | Upregulated |
|  |  | **miR-33b** | Upregulated |
|  |  | **miR-99a** | Upregulated |
|  |  | **miR-124** | Upregulated |
|  |  | **miR-133a** | Downregulated |
|  |  | **miR-133b** | Downregulated |
|  |  | **miR-133c** | Downregulated |
|  |  | **miR-134** | Downregulated |
|  |  | **miR-135a** | Upregulated |
|  |  | **miR-187** | Upregulated |
|  |  | **miR-200a** | Upregulated |
|  |  | **miR-202** | Upregulated |
|  |  | **miR-206** | Downregulated |
|  |  | **miR-208b** | Downregulated |
|  |  | **miR-212** | Upregulated |
|  |  | **miR-323** | Downregulated |
|  |  | **miR-370** | Downregulated |
|  |  | **miR-380** | Downregulated |
|  |  | **miR-383** | Downregulated |
|  |  | **miR-424** | Upregulated |
|  |  | **miR-432** | Downregulated |
|  |  | **miR-450a** | Upregulated |
|  |  | **miR-450b** | Upregulated |
|  |  | **miR-485**^1^ | Downregulated |
|  |  | **miR-486** | Downregulated |
|  |  | **miR-488** | Downregulated |
|  |  | **miR-503** | Upregulated |
|  |  | **miR-504** | Downregulated |
|  |  | **miR-539** | Downregulated |
|  |  | **miR-542** | Upregulated |
|  |  | **miR-551b** | Downregulated |
|  |  | **miR-592** | Upregulated |
|  |  | **miR-1836** | Upregulated |
|  |  | **miR-2387** | Upregulated |
| Mammary carcinoma | Mammary tumor cells |  |  |
|  |  | **Let-7b** (7) | Not reported |
|  |  | **Let-7d** (7) | Upregulated |
|  |  | **Let-7f** (70, 123) | Upregulated |
|  |  | **Let-7f** (58) | Downregulated |
|  |  | **Let-7g** (41, 123) | Upregulated |
|  |  | **miR-1** (30, 35, 41) | Downregulated |
|  |  | **miR-7** (35, 41) | Upregulated |
|  |  | **miR-9** (7, 30, 35) | Upregulated |
|  |  | **miR-10a** (7, 35, 41) | Downregulated |
|  |  | **miR-10b** (58, 123) | Upregulated |
|  |  | **miR-15 (**119) | Downregulated |
|  |  | **miR-15a** (7, 41) | Upregulated |
|  |  | **miR-15a** (58, 70) | Downregulated |
|  |  | **miR-15b** (7, 41) | Upregulated |
|  |  | **miR-15b** (70) | Downregulated |
|  |  | **miR-16** (7, 41) | Upregulated |
|  |  | **miR-16** (70) | Downregulated |
|  |  | **miR-16** (119) | Downregulated |
|  |  | **miR-17** (35) | Upregulated |
|  |  | **miR-17** (70) | Downregulated |
|  |  | **miR-18a** (7,41) | Upregulated |
|  |  | **miR-18b** (41) | Upregulated |
|  |  | **miR-19a** (35, 41) | Upregulated |
|  |  | **miR-19a** (58) | Downregulated |
|  |  | **miR-20a** (7,41) | Upregulated |
|  |  | **miR-20b** (41) | Upregulated |
|  |  | **miR-21** (35, 41, 70) | Upregulated |
|  |  | **miR-21** 58) | Upregulated |
|  |  | **miR-23** (7,41) | Upregulated |
|  |  | **miR-23b** (7,41) | Upregulated |
|  |  | **miR-24** (41) | Upregulated |
|  |  | **miR-25** (35) | Upregulated |
|  |  | **miR-26b** (58) | Downregulated |
|  |  | **miR-19b** (7, 35, 41) | Upregulated |
|  |  | **miR-22** (41) | Upregulated |
|  |  | **miR-26a** (41) | Downregulated |
|  |  | **miR-27a** (41) | Upregulated |
|  |  | **miR-27b** (41) | Upregulated |
|  |  | **miR-29a** (41) | Upregulated |
|  |  | **miR-29a (58)** | Downregulated |
|  |  | **miR-29b** (7, 35, 41, 58, 70, 123) | Upregulated |
|  |  | **miR-29c** (7, 41) | Upregulated |
|  |  | **miR-30** (41) | Upregulated |
|  |  | **miR-30** (58) | Downregulated |
|  |  | **miR-30b** (7, 41) | Upregulated |
|  |  | **miR-30c (**41, 58) | Downregulated |
|  |  | **miR-30d** (41) | Upregulated |
|  |  | **miR-30e** (41) | Downregulated |
|  |  | **miR-31** (35, 41) | Upregulated/Downregulated |
|  |  | **miR-32** (7,41) | Upregulated |
|  |  | **miR-33a** (35) | Upregulated |
|  |  | **miR-34a** (24, 41) | Downregulated |
|  |  | **miR-34a** (35) | Upregulated/Downregulated |
|  |  | **miR-34b** (7,41) | Upregulated |
|  |  | **miR-71** (35, 41) | Upregulated |
|  |  | **miR-93** (41) | Upregulated |
|  |  | **miR-93** (35) | Upregulated |
|  |  | **miR-95** (35,41) | Upregulated |
|  |  | **miR-98** (41) | Downregulated |
|  |  | **miR-99a** (7) | Upregulated |
|  |  | **miR-99a** (30) | Downregulated |
|  |  | **miR-99b** (35, 41) | Upregulated |
|  |  | **miR-101** (7, 35, 41) | Upregulated |
|  |  | **miR-101** (58) | Downregulated |
|  |  | **miR-103b** (35, 41) | Downregulated |
|  |  | **miR-106a** (7, 35, 41) | Upregulated |
|  |  | **miR-106b** (7, 41) | Upregulated |
|  |  | **miR-122** (7, 41) | Upregulated |
|  |  | **miR-122** (35) | Upregulated/Downregulated |
|  |  | **miR-124** (107) | Downregulated |
|  |  | **miR-124a** (7, 41) | Upregulated |
|  |  | **miR-125a** (58) | Downregulated |
|  |  | **miR-125b** (41) | Upregulated |
|  |  | **miR-125b** (58, 70) | Downregulated |
|  |  | **miR-126** (35, 41) | Upregulated |
|  |  | **miR-126** (7, 35, 41) | Downregulated |
|  |  | **miR-128** (35) | Upregulated/Downregulated |
|  |  | **miR-130** (35) | Upregulated |
|  |  | **miR-130a** (35) | Upregulated |
|  |  | **miR-133** (7, 30, 35) | Downregulated |
|  |  | **miR-133a** (35) | Downregulated |
|  |  | **miR-133b** (30) | Downregulated |
|  |  | **miR-133c** (30) | Downregulated |
|  |  | **miR-135a** (41) | Upregulated |
|  |  | **miR-136** (58) | Downregulated |
|  |  | **miR-137** (35, 41) | Upregulated |
|  |  | **miR-138a** (123) | Downregulated |
|  |  | **miR-138a** (35) | Upregulated/Downregulated |
|  |  | **miR-139** (7, 35) | Upregulated |
|  |  | **miR-143** (78) | Downregulated |
|  |  | **miR-145** (58, 78) | Downregulated |
|  |  | **miR-146a** (35, 41) | Upregulated/Downregulated |
|  |  | **miR-147** (41) | Upregulated |
|  |  | **miR-148a** (41, 58) | Downregulated |
|  |  | **miR-148a** (35, 41) | Upregulated |
|  |  | **miR-148b** (58) | Downregulated |
|  |  | **miR-148b** (35) | Upregulated/Downregulated |
|  |  | **miR-150** (35) | Upregulated |
|  |  | **miR-181a** (35, 41, 70) | Upregulated |
|  |  | **miR-130b** (7) | Upregulated |
|  |  | **miR-133b** (7, 35) | Downregulated |
|  |  | **miR-134** (7) | Upregulated |
|  |  | **miR-140** (35) | Downregulated |
|  |  | **miR-140** (41) | Upregulated |
|  |  | **miR-141** (58) | Downregulated |
|  |  | **miR-142** (35, 41 78) | Downregulated |
|  |  | **miR-143** (41, 123) | Upregulated |
|  |  | **miR-144** (35, 41) | Downregulated |
|  |  | **miR-144** (41, 78, 123) | Upregulated |
|  |  | **miR-151** (35, 41) | Upregulated/Downregulated |
|  |  | **miR-153** (7, 35, 41) | Upregulated |
|  |  | **miR-155** (70) | Downregulated |
|  |  | **miR-181a** (58) | Downregulated |
|  |  | **miR-181b** (7, 41) | Upregulated |
|  |  | **miR-181c** (35, 41) | Upregulated |
|  |  | **miR-181d** (7, 35, 41) | Upregulated |
|  |  | **miR-182** (7, 35, 41) | Upregulated |
|  |  | **miR-184** (35, 41) | Upregulated |
|  |  | **miR-185** (35, 41) | Upregulated |
|  |  | **miR-188** (35) | Downregulated |
|  |  | **miR-190a** (7, 35)^40,89^ | Upregulated |
|  |  | **miR-192** (41) | Upregulated |
|  |  |  |  |
|  |  |  |  |
|  |  |  |  |
|  |  |  |  |
|  |  |  |  |
|  |  | **miR-194** (58) | Downregulated |
|  |  | **miR-195** (58) | Upregulated |
|  |  | **miR-196a** (41, 58) | Downregulated |
|  |  | **miR-196b** (30) | Downregulated |
|  |  | **miR-196b** (7) | Upregulated |
|  |  | **miR-198** (7, 35, 41) | Upregulated/Downregulated |
|  |  | **miR-200** (35, 41) | Upregulated |
|  |  | **miR-200a** (41) | Upregulated |
|  |  | **miR-200b** (7, 35, 41) | Upregulated/Downregulated |
|  |  | **miR-203** (7, 35, 41) | Upregulated |
|  |  | **miR-204** (35, 41) | Upregulated/Downregulated |
|  |  | **miR-205** (35, 41) | Downregulated |
|  |  | **miR-206** (30) | Downregulated |
|  |  | **miR-208** (30) | Downregulated |
|  |  | **miR-210** (24, 58, 78) | Upregulated |
|  |  | **miR-210** (35) | Upregulated/Downregulated |
|  |  | **miR-211** (41) | Upregulated |
|  |  | **miR-212** (35, 41) | Downregulated |
|  |  | **miR-214** (7, 41) | Upregulated |
|  |  | **miR-216b** (7, 35) | Upregulated/Downregulated |
|  |  | **miR-218** (7) | Upregulated |
|  |  | **miR-222** (41) | Downregulated |
|  |  | **miR-222** (7, 35) | Upregulated/Downregulated |
|  |  | **miR-223** (35) | Downregulated |
|  |  | **miR-229** (35) | Upregulated/Downregulated |
|  |  | **miR-302d** (58) | Downregulated |
|  |  | **miR-323** (41) | Upregulated |
|  |  | **miR-324** (35) | Upregulated/Downregulated |
|  |  | **miR-329b** (35, 41) | Upregulated |
|  |  | **miR-330** (7, 41) | Upregulated |
|  |  | **miR-331** (35, 41) | Upregulated/Downregulated |
|  |  | **miR-335** (7, 41) | Upregulated |
|  |  | **miR-350** (7) | Upregulated |
|  |  | **miR-338** (30) | Downregulated |
|  |  | **miR-339** (41) | Upregulated |
|  |  | **miR-342** (7, 35, 41) | Upregulated |
|  |  | **miR-345** (7) | Upregulated |
|  |  | **miR-362** (35, 41) | Upregulated/Downregulated |
|  |  | **miR-365** (7) | Downregulated |
|  |  | **miR-365** (35, 41) | Upregulated |
|  |  | **miR-373** (7) | Upregulated |
|  |  | **miR-374a** (58) | Downregulated |
|  |  | **miR-374a** (7, 41) | Upregulated |
|  |  | **miR-375** (35) | Downregulated |
|  |  | **miR-376a** (41) | Upregulated |
|  |  | **miR-376c** (35) | Downregulated |
|  |  | **miR-377** (30) | Downregulated |
|  |  | **miR-378b** (35, 41) | Upregulated/Downregulated |
|  |  | **miR-379** (30) | Downregulated |
|  |  | **miR-380** (41) | Upregulated |
|  |  | **miR-380** (35) | Upregulated/Downregulated |
|  |  | **miR-381** (41) | Upregulated |
|  |  | **miR-382** (41) | Downregulated |
|  |  | **miR-409** (41) | Upregulated |
|  |  | **miR-410** (35) | Upregulated/Downregulated |
|  |  | **miR-411** (7, 41) | Upregulated |
|  |  | **miR-424** (7, 41) | Upregulated |
|  |  | **miR-425** (35, 41) | Upregulated |
|  |  | **miR-429** (35) | Downregulated |
|  |  | **miR-452** (35) | Downregulated |
|  |  | **miR-452** (41) | Upregulated |
|  |  | **miR-454** (41) | Downregulated |
|  |  | **miR-455** (41) | Upregulated |
|  |  | **miR-485** (30) | Downregulated |
|  |  | **miR-485** (7, 41) | Upregulated |
|  |  | **miR-490** (41) | Downregulated |
|  |  | **miR-491** (35, 41 58) | Upregulated/Downregulated |
|  |  | **miR-494** (35, 41) | Downregulated |
|  |  | **miR-495** (35, 41) | Upregulated |
|  |  | **miR-497** (119) | Downregulated |
|  |  | **miR-500** (41) | Upregulated |
|  |  | **miR-500a** (35, 41) | Upregulated |
|  |  | **miR-503** (7, 35, 41) | Upregulated |
|  |  | **miR-504** (41) | Upregulated |
|  |  | **miR-505** (35, 41) | Upregulated |
|  |  | **miR-542** (35) | Upregulated/Downregulated |
|  |  | **miR-542** (41) | Upregulated |
|  |  | **miR-548c** (7) | Upregulated |
|  |  | **miR-551b** (35, 41) | Downregulated |
|  |  | **miR-590** (35, 41) | Upregulated |
|  |  | **miR-615** (41) | Upregulated |
|  |  | **miR-615** (35) | Upregulated/Downregulated |
|  |  | **miR-653** (41)^39^ | Upregulated |
|  |  | **miR-660** (41)^39^ | Upregulated |
|  |  | **miR-675** (41) | Downregulated |
|  |  | **miR-758** (41) | Upregulated |
|  |  | **miR-874** (41) | Upregulated |
|  |  | **miR-889** (41) | Downregulated |
|  |  | **miR-1197** (41) | Upregulated |
|  |  | **miR-1271** (30) | Downregulated |
|  |  | **miR-1296** (41) | Upregulated |
|  |  | **miR-1301** (41) | Upregulated |
|  |  | **miR-1307** (41) | Upregulated |
|  |  | **miR-1346** (7, 41) | Upregulated |
|  |  | **miR-1839** (41) | Upregulated |
|  |  | **miR-1841** (7) | Upregulated |
|  |  | **miR-1842** (41) | Upregulated |
|  |  | **miR-4859** (7, 41) | Upregulated |
|  |  | **miR-7638** (41) | Upregulated |
|  |  | **miR-8859a** (7) | Upregulated |
|  |  | **miR-8865** (41) | Downregulated |
|  |  | **miR-8865** (7)^40^ | Upregulated |
|  |  | **miR-8884** (41) | Downregulated |
|  |  |  |  |
|  | PBMCs (91) | **miR-96** | Upregulated |
|  |  | **miR-149** | Downregulated |
|  |  | **miR-8832** | Upregulated |
| Mammary tumor | Serum (16) | **miR-21** | Upregulated |
| Metastasis to mammary tissues (78) | Mammary tissue | **miR-101** | Upregulated |
|  |  | **miR-29b** | Upregulated |
| Mast Cell Tumor | Mast Cell tumor vs. adjacent tissue | **Let-7g** (67) | Upregulated |
|  |  | **miR-9** 85) | Upregulated |
|  |  | **miR-17** (67) | Downregulated |
|  |  | **miR-17** (85) | Upregulated |
|  |  | **miR-18a** (85) | Upregulated |
|  |  | **miR-18b** (85) | Upregulated |
|  |  | **miR-19a** (85) | Upregulated |
|  |  | **miR-20b** (67) | Downregulated |
|  |  | **miR-20b** (85) | Upregulated |
|  |  | **miR-21** (67) | Upregulated |
|  |  | **miR-23a** (67) | Downregulated |
|  |  | **miR-25** (85) | Upregulated |
|  |  | **miR-26a** (67) | Downregulated |
|  |  | **miR-92a** (67) | Downregulated |
|  |  | **miR-93** (85) | Upregulated |
|  |  | **miR-95** (67) | Downregulated |
|  |  | **miR-105a** (67) | Downregulated |
|  |  | **miR-106a** (67) | Downregulated |
|  |  | **miR-106a** (85) | Upregulated |
|  |  | **miR-130a** (67) | Downregulated |
|  |  | **miR-133a** (67) | Downregulated |
|  |  | **miR-141** (67) | Downregulated |
|  |  | **miR-142** (67) | Upregulated |
|  |  | **miR-142** (85) | Upregulated |
|  |  | **miR-145** (67) | Downregulated |
|  |  | **miR-149** (67) | Downregulated |
|  |  | **miR-153** (67) | Upregulated |
|  |  | **miR-147** (67) | Upregulated |
|  |  | **miR-31** (67) | Downregulated |
|  |  | **miR-96** (67) | Downregulated |
|  |  | **miR-106b** (85) | Upregulated |
|  |  | **miR-130b** (85) | Upregulated |
|  |  | **miR-133c** (67) | Downregulated |
|  |  | **miR-138** (85) | Upregulated |
|  |  | **miR-148b** (85) | Upregulated |
|  |  | **miR-181d** (67) | Downregulated |
|  |  | **miR-182** (67) | Downregulated |
|  |  | **miR-182** (85) | Upregulated |
|  |  | **miR-183** (67) | Downregulated |
|  |  | **miR-184** (67) | Downregulated |
|  |  | **miR-186** (85) | Upregulated |
|  |  | **miR-187** (67) | Downregulated |
|  |  | **miR-187** (85) | Upregulated |
|  |  | **miR-191** (85) | Upregulated |
|  |  | **miR-192** (85) | Upregulated |
|  |  | **miR-193a** (85) | Upregulated |
|  |  | **miR-194** (85) | Upregulated |
|  |  | **miR-197** (67) | Downregulated |
|  |  | **miR-200a** (67) | Downregulated |
|  |  | **miR-200a** (85) | Downregulated |
|  |  | **miR-200b** (67) | Downregulated |
|  |  | **miR-200c** (67) | Downregulated |
|  |  | **miR-203** (67) | Downregulated |
|  |  | **miR-211** (67) | Downregulated |
|  |  | **miR-212** (67) | Upregulated |
|  |  | **miR-204** (67) | Downregulated |
|  |  | **miR-205** (67) | Downregulated |
|  |  | **miR-299** (67) | Downregulated |
|  |  | **miR-299** (85) | Downregulated |
|  |  | **miR-301a** (67) | Downregulated |
|  |  | **miR-301a** (85) | Upregulated |
|  |  | **miR-301b** (85) | Upregulated |
|  |  | **miR-302b** (85) | Upregulated |
|  |  | **miR-214** (67) | Downregulated |
|  |  | **miR-216b** (85) | Upregulated |
|  |  | **miR-217** (85) | Upregulated |
|  |  | **miR-324** (85) | Upregulated |
|  |  | **miR-326** (85) | Upregulated |
|  |  | **miR-330** (85) | Upregulated |
|  |  | **miR-338** (67) | Downregulated |
|  |  | **miR-342** (67) | Downregulated |
|  |  | **miR-363** (67) | Downregulated |
|  |  | **miR-370** (67) | Upregulated |
|  |  | **miR-372** (85) | Upregulated |
|  |  | **miR-375** (67) | Downregulated |
|  |  | **miR-376c** (67) | Upregulated |
|  |  | **miR-379** (67) | Upregulated |
|  |  | **miR-409** (67) | Upregulated |
|  |  | **miR-411** (67) | Upregulated |
|  |  | **miR-412** (85) | Downregulated |
|  |  | **miR-421** (67) | Downregulated |
|  |  | **miR-423** (85) | Upregulated |
|  |  | **miR-425** (85) | Upregulated |
|  |  | **miR-429** (67) | Downregulated |
|  |  | **miR-448** (85) | Upregulated |
|  |  | **miR-449b** (85) | Upregulated |
|  |  | **miR-450a** (67) | Upregulated |
|  |  | **miR-451** (85) | Upregulated |
|  |  | **miR-454** (85) | Upregulated |
|  |  | **miR-486** (67) | Downregulated |
|  |  | **miR-486** (85) | Downregulated |
|  |  | **miR-488** (85) | Downregulated |
|  |  | **miR-493** (67) | Upregulated |
|  |  | **miR-518f** (85) | Upregulated |
|  |  | **miR-520b** (85) | Upregulated |
|  |  | **miR-548b** (85) | Upregulated |
|  |  | **miR-618** (85) | Upregulated |
|  |  | **miR-632** (67) | Upregulated |
|  |  | **miR-636** (85) | Upregulated |
|  |  | **miR-652** (85) | Upregulated |
|  |  | **miR-664** (67) | Upregulated |
|  |  | **miR-708** (67) | Downregulated |
|  |  | **miR-872** (85) | Upregulated |
|  |  | **miR-874** (85) | Downregulated |
|  |  | **miR-885** (67) | Downregulated |
|  |  | **miR-885** (85) | Downregulated |
|  |  | **miR-1217** (67) | Downregulated |
|  |  | **miR-1296** (67) | Downregulated |
|  |  | **miR-1306** (67) | Downregulated |
|  |  | **miR-1343** (67) | Downregulated |
|  |  | **miR-8884** (67) | Upregulated |
|  |  | **miR-8904b** (67) | Upregulated |
| Malignant melanoma (66) | Melanoma | **miR-9** | Upregulated |
|  |  | **miR-10** | Downregulated |
|  |  | **miR-101** | Downregulated |
|  |  | **miR-122** | Downregulated |
|  |  | **miR-142** | Downregulated |
|  |  | **miR-143** | Downregulated |
|  |  | **miR-149** | Upregulated |
|  |  | **miR-195** | Downregulated |
|  |  | **miR-200c** | Downregulated |
|  |  | **miR-205** | Upregulated |
|  |  | **miR-326** | Upregulated |
|  |  | **miR-328** | Downregulated |
|  |  | **miR-383** | Upregulated |
|  |  | **miR-487b** | Downregulated |
|  |  | **miR-652** | Downregulated |
| Primary melanoma (137) | Melanoma cell lines | **Let-7b** | Upregulated |
|  |  | **miR-143** | Upregulated |
| Melanoma | Melanoma cells | **miR-146b** (136) | Upregulated |
|  |  | **miR-203** (99, 131) | Downregulated |
|  |  | **miR-205** (24) | Downregulated |
|  |  | **miR-210** (136) | Upregulated |
|  |  | **miR-301a** (136) | Upregulated |
|  |  | **miR-450b** (136) | Upregulated |
| Cutaneous malignant melanoma (52) | Cutaneous melanoma cells | **miR-146a** | Upregulated |
|  | Cutaneous melanoma (FFPE) | **miR-425** | Downregulated |
| Oral melanoma | Oral melanoma | **Let-7a** (65) | Downregulated |
|  |  | **Let-7b** (65) | Downregulated |
|  |  | **miR-9** (65) | Upregulated |
|  |  | **miR-20b** (65) | Upregulated |
|  |  | **miR-21** (65) | Upregulated |
|  |  | **miR-26b** (65) | Downregulated |
|  |  | **miR-29c** (65) | Downregulated |
|  |  | **miR-31** (65) | Downregulated |
|  |  | **miR-101** (65) | Downregulated |
|  |  | **miR-125a** (65) | Downregulated |
|  |  | **miR-126** (52,65) | Downregulated |
|  |  | **miR-126** (64) | Downregulated |
|  |  | **miR-129** (65) | Upregulated |
|  |  | **miR-132** (65) | Upregulated |
|  |  | **miR-141** (65) | Downregulated |
|  |  | **miR-145** (65) | Upregulated |
|  |  | **miR-146b** (65) | Upregulated |
|  |  | **miR-152** (65) | Downregulated |
|  |  | **miR-183** (65) | Downregulated |
|  |  | **miR-200** (65) | Downregulated |
|  |  | **miR-200a** (52, 64) | Downregulated |
|  |  | **miR-203** (52, 64, 65) | Downregulated |
|  |  | **miR-205** (52, 64, 65) | Downregulated |
|  |  | **miR-223** (65) | Upregulated |
|  |  | **miR-300** (65) | Upregulated |
|  |  | **miR-335** (65) | Upregulated |
|  |  | **miR-383** (65) | Upregulated |
|  |  | **miR-423** (65) | Upregulated |
|  |  | **miR-423a** (65) | Upregulated |
|  |  | **miR-449** (65) | Upregulated |
|  |  | **miR-454** (65) | Upregulated |
|  |  | **miR-708** (52) | Downregulated |
|  |  | **miR-514** (52) | Upregulated |
|  |  | **miR-517b** (64) | Downregulated |
|  |  | **miR-520c** (52) | Downregulated |
|  |  | **miR-520c** (64) | Upregulated |
|  |  | **miR-713** (64) | Downregulated |
| Melanoma (24, 101) | Malignant melanoma cells | **miR-145** | Downregulated |
| Metastatic melanoma (137) | Melanoma cell lines | **miR-210** | Upregulated |
|  |  | **miR-221** | Upregulated |
|  |  | **miR-222** | Upregulated |
|  |  | **miR-708** | Upregulated |
| Uveal melanoma (metastasizing tumors) (160) | Uveal melanoma | **miR-124** | Upregulated |
|  |  | **miR-130b** | Upregulated |
|  |  | **miR-155** | Upregulated |
|  |  | **miR-182** | Upregulated |
|  |  | **miR-362** | Upregulated |
|  |  | **miR-500** | Upregulated |
|  |  | **miR-543** | Upregulated |
|  |  | **miR-2287** | Downregulated |
|  |  | **miR-2382** | Downregulated |
|  |  | **miR-2387** | Downregulated |
|  |  | **miR-2398** | Downregulated |
|  |  | **miR-2411** | Upregulated |
|  |  | **miR-4430** | Upregulated |
|  |  | **miR-4531** | Upregulated |
| Metastatic Melanoma (137) | Serum | **miR-143** | Upregulated |
|  |  | **miR-221** | Upregulated |
| Osteosarcoma | Bone (FFT) | **miR-BART11** (31) | Downregulated |
|  |  | **miR-1** (31, 34) | Downregulated |
|  |  | **miR-9** (31) | Upregulated |
|  |  | **miR-16** (31) | Upregulated |
|  |  | **miR-19a** (31) | Upregulated |
|  |  | **miR-22** (31) | Downregulated |
|  |  | **miR-25** (31) | Upregulated |
|  |  | **miR-26b** (31) | Upregulated |
|  |  | **miR-29a** (31) | Downregulated |
|  |  | **miR-29b**^38^ | Downregulated |
|  |  | **miR-30e** (31) | Upregulated |
|  |  | **miR-34a** (31) | Downregulated |
|  |  | **miR-34a** (140) | Downregulated |
|  |  | **miR-92a** (161) | Upregulated |
|  |  | **miR-98** (31) | Downregulated |
|  |  | **miR-125a** (31) | Downregulated |
|  |  | **miR-126** (31) | Upregulated |
|  |  | **miR-133** (31) | Downregulated |
|  |  | **miR-133b** (34) | Downregulated |
|  |  | **miR-142** (31) | Upregulated |
|  |  | **miR-143** (31) | Downregulated |
|  |  | **miR-144** (31) | Upregulated |
|  |  | **miR-145** (31) | Downregulated |
|  |  | **miR-148b** (31) | Downregulated |
|  |  | **miR-181c** (31) | Upregulated |
|  |  | **miR-192** (31) | Downregulated |
|  |  | **miR-193a** (31) | Downregulated |
|  |  | **miR-193b** (31) | Downregulated |
|  |  | **miR-195** (31) | Upregulated |
|  |  | **miR-196a** (104) | Downregulated |
|  |  | **miR-197** (31) | Upregulated |
|  |  | **miR-199b** (31) | Upregulated |
|  |  | **miR-200a** (31) | Downregulated |
|  |  | **miR-342** (31) | Upregulated |
|  |  | **miR-346** (31) | Downregulated |
|  |  | **miR-362** (31) | Upregulated |
|  |  | **miR-365** (31) | Downregulated |
|  |  | **miR-423** (31) | Upregulated |
|  |  | **miR-450a** (31) | Upregulated |
|  |  | **miR-451** (31) | Upregulated |
|  |  | **miR-490** (31) | Downregulated |
|  |  | **miR-497** (31) | Upregulated |
|  |  | **miR-499** (31) | Upregulated |
|  |  | **miR-506** (31) | Downregulated |
|  |  | **miR-518d** (31) | Downregulated |
|  |  | **miR-518f** (31) | Downregulated |
|  |  | **miR-520c** (31) | Downregulated |
|  |  | **miR-523** (31) | Downregulated |
|  |  | **miR-526a** (31) | Downregulated |
|  |  | **miR-532** (31) | Upregulated |
|  |  | **miR-539** (31) | Downregulated |
|  |  | **miR-579** (31) | Downregulated |
|  |  | **miR-592** (31) | Upregulated |
|  |  | **miR-600** (31) | Downregulated |
|  |  | **miR-617** (31) | Downregulated |
|  |  | **miR-640** (31) | Downregulated |
|  |  | **miR-655** (31) | Downregulated |
|  |  | **miR-664** (31) | Downregulated |
|  |  | **miR-744** (31) | Downregulated |
|  |  | **miR-770** (31) | Downregulated |
|  |  | **miR-892a** (31) | Downregulated |
|  |  | **miR-943** (31) | Downregulated |
|  |  | **miR-1178** (31) | Downregulated |
|  |  | **miR-1179** (31) | Downregulated |
|  |  | **miR-1206** (31) | Downregulated |
|  |  | **miR-1225** (31) | Upregulated |
|  |  | **miR-1255a** (31) | Downregulated |
|  |  | **miR-1262** (31) | Downregulated |
|  |  | **miR-1274b** (31) | Downregulated |
|  |  | **miR-1275** (31) | Downregulated |
|  |  | **miR-1277** (31) | Downregulated |
|  |  | **miR-1279** (31) | Downregulated |
|  |  | **miR-1283** (31) | Downregulated |
|  | Bone (FFPE) | **miR-1** (34) | Downregulated |
|  |  | **miR-93** (161) | Upregulated |
|  | Osteosarcoma cell lines (140) | **miR-34a** | Downregulated |
| Appendicular Osteosarcoma (49) | Plasma | **miR-126** | Upregulated |
|  |  | **miR-214** | Upregulated |
| Grade III (compared to grade II) osteosarcoma (48) | Plasma | **miR-126** | Upregulated |
|  |  | **miR-214** | Upregulated |
| Prostate cancer | CT1258-EGFP and CT1258-EGFP-HMGA2 cell lines (116) | **Let-7a** | Upregulated |
|  | Prostatic tissue (24, 60) | **miR-18a** | Upregulated |
|  |  | **miR-221** | Upregulated |
| Prostatic carcinoma (60) | Prostatic tissue | **miR-95** | Upregulated |
|  |  | **miR-127** | Downregulated |
|  |  | **miR-146a** | Upregulated |
|  |  | **miR-148a** | Downregulated |
|  |  | **miR-205** | Downregulated |
|  |  | **miR-229** | Downregulated |
|  |  | **miR-329b** | Downregulated |
|  |  | **miR-330** | Upregulated |
|  |  | **miR-335** | Downregulated |
|  |  | **miR-376a** | Downregulated |
|  |  | **miR-379** | Downregulated |
|  |  | **miR-380** | Downregulated |
|  |  | **miR-381** | Downregulated |
|  |  | **miR-411** | Downregulated |
|  |  | **miR-487b** | Downregulated |
|  |  | **miR-495** | Downregulated |
| Oral squamous cell carcinoma (113) | Oral squamous cell carcinoma tissue | **Let-7a** | Upregulated |
|  |  | **miR-98** | Upregulated |
| Squamous cell carcinoma (102) | Oral mucosal tissue | **miR-145** | Downregulated |
| Urothelial carcinoma | FFPE urinary bladder (114) | **miR-16** | Upregulated |
|  |  | **miR-34a** | Upregulated |
|  |  | **miR-103a** | Upregulated |
|  |  | **miR-106b** | Upregulated |
|  | Urothelium (51) | **miR-7** | Downregulated |
|  |  | **miR-32** | Downregulated |
|  |  | **miR-99a** | Downregulated |
|  |  | **miR-105a** | Downregulated |
|  |  | **miR-143** | Downregulated |
|  |  | **miR-145** | Downregulated |
|  |  | **miR-181a** | Downregulated |
|  |  | **miR-190a** | Downregulated |
|  |  | **miR-214** | Downregulated |
|  |  | **miR-216a** | Downregulated |
|  |  | **miR-223** | Downregulated |
|  |  | **miR-301a** | Downregulated |
|  |  | **miR-329b** | Downregulated |
|  |  | **miR-361** | Downregulated |
|  |  | **miR-374b** | Upregulated |
|  |  | **miR-429** | Upregulated |
|  |  | **miR-450a** | Downregulated |
|  |  | **miR-490** | Downregulated |
|  |  | **miR-532** | Downregulated |
|  |  | **miR-551b** | Downregulated |
|  |  | **miR-544** | Downregulated |
|  |  | **miR-568** | Downregulated |
|  |  | **miR-582** | Downregulated |
|  |  | **miR-652** | Downregulated |
|  |  | **miR-764** | Downregulated |
|  |  | **miR-802** | Downregulated |
|  |  | **miR-874** | Downregulated |
|  |  | **miR-876** | Downregulated |
|  | Urine (10) | **miR-16** | Downregulated |
|  |  | **miR-103b** | Downregulated |
|  |  | **miR-106b** | Downregulated |
|  | Whole blood (10) | **miR-103b** | Downregulated |
