# Supplementary material for: A review on microRNA detection and expression studies in dogs
Source: Front Vet Sci. 2023 Oct 5;10:1261085. doi: 10.3389/fvets.2023.1261085 (PMC10585042; doi:10.3389/fvets.2023.1261085)
Supplement: Supplementary file 1 [file Data_Sheet_1.zip › Table S5.DOCX]

**Table S5**. Reported target, pathways of action, and function of miRNA in different disease processes in dogs. MiRNAs expressed in all four of the groups considered (normal processes, noninflammatory/noninfectious processes, inflammatory/infectious processes, and neoplasia) are highlighted.

| **miRNA** | **Target** | **Pathway affected** | **miRNA function** | **Disease process** | **References** |
| --- | --- | --- | --- | --- | --- |
| **miR-1** | MET |  | Inhibits cell proliferation in a MET dependent manner | Hepatocellular Carcinoma | (33) |
|  | EDFR |  |  | Mammary carcinoma | (35,41) |
| **miR-10b** | BUB1, PLK1, CCNA2 | Cell cycle regulation |  | Normal tissue>benign mammary tumors>mammary carcinoma | (58) |
| **miR cluster 14q32** |  | Regulates c-Myc expression | Decrease metastasis and increase survival times | Osteosarcoma | (24) |
| **miR-15a** | CCNE1 | Cell cycle regulation |  |  | (58, 70) |
| **miR-17** | TGF-Beta R1/2 |  | EMT, fibrosis | Myxomatous mitral valve disease | (45) |
| **miR-19a** | Fra-1 proto-oncogene | Induce macrophage polarization |  |  | (58) |
| **miR-20a** | TGF-Beta R1/2 |  | EMT, fibrosis | Myxomatous mitral valve disease | (45) |
| **miR-21** | SMAD7 | TGF-β signaling pathway | Inhibits the TGF-β /Smad2/3 pathway | XLHN | (75) |
|  |  | PTEN, TPM1 | Overexpression | Mammary carcinoma | (24, 35, 41, 70) |
|  |  | PTEN/PI3K/Akt pathway, HIF-1a pathway |  | Bone regeneration in bone defects | (118) |
|  | CD69 |  |  |  | (5) |
| **miR-22** |  | Downregulation of PTEN |  | hemangiosarcoma | (127) |
| **miR-23a-3p** |  | Inhibit the transcription of SLC7A11 mRNA and depleting the Xc^-^ transport system | Promote ferroptosis of cardiomyocytes | Atrial fibrillation | (148) |
| **miR-25** | P57/Kip2 |  |  | Mammary carcinoma | (2) |
| **miR-26a** | VEGFA |  | Angiogenesis | hemangiosarcoma | (127) |
|  | Atrial fibroblast inward-rectifier K+ current |  |  | Congestive Heart Failure | (105) |
| **miR-26b** | SLC7A11 |  | Apoptosis | Mammary carcinoma | (58) |
| **miR-27a** | DDK2, SFRP1 |  | Enhances osteogenesis and Angiogenesis | Peri-implantitis | (117) |
| **miR-29a** | Col4a2, Spry1, Timp3 | Antiangiogenic |  | Mammary carcinoma | (58) |
| **miR-29b** | COL1A1, COL3A1 |  |  | Atrial fibrillation | (77) |
| **miR-30a** | MTDH | Angiogenesis |  | Mammary carcinoma | (58) |
| **miR-30c** | KRAS | Signaling |  | Mammary carcinoma | (41, 58) |
| **miR-30d** | MAP4K4 |  |  | Myxomatous mitral valve disease | (45) |
| **miR-34** | KLF4, VEGFA |  |  | Osteosarcoma | (140) |
| **miR-122** |  |  | Hepatocyte derived miRNA – positive correlation with grade of hepatitis and stage of fibrosis | Acute and Chronic hepatitis | (110, 158) |
| **miR-124** |  | Regulates p53 via COP1 E3 ubiquitin-protein ligase |  | Hemangiosarcoma | (24) |
| **miR-125a** | HER2, HER3 | Epidermal growth factor receptors |  | Metastatic mammary carcinoma compared with benign mammary tumors | (58) |
| **miR-125b** | HER2, HER3 | Epidermal growth factor receptors |  | Mammary tumors | (58) |
|  | PI3K regulator subunit 2, RGS16 | Increases VEGF expression, inhibits CXCR4 | Angiogenesis – both a pro and anti-angiogenic factor | hemangiosarcoma | (58) |
| **miR-126** | P27//Kip1 |  |  | Mammary carcinoma | (35,41) |
| **miR-132** |  | Repression of TGF-Beta1 |  | Nicotine-induced atrial remodeling | (111) |
|  | Connective tissue growth factor |  |  | Atrial fibrillation | (106) |
| **miR-133b** |  | NK cell mediate cytotoxicity, Notch signaling |  | H5N1, H3N2 | (145) |
| **miR-138b** |  | CXCR4 | Angiogenesis | Hemangiosarcoma | (127) |
| **miR-139** | VEGFA |  | Angiogenesis | Hemangiosarcoma | (127) |
| **miR-140** | P16/INK4A, p14ARF |  |  | Mammary carcinoma | (41) |
| **miR-143** | Igfbp5 gene | P53 pathway | Apoptosis | Canine Influenza Virus | (120) |
|  | ERK5 |  |  | Canine Influenza Virus | (144) |
| **miR-145** |  | Inhibits cell grown via c-Myc and reduces cell migration via FASCIN1 | Tumor suppressor | Melanoma | (24, 101) |
| **miR-148a** | ERBB3 | Growth factor |  | Mammary carcinoma | (41, 58) |
| **miR-148b** | ITGA5, ROCK1, PIK3CA, NRAS, CSF1 |  |  | Mammary carcinoma | (58) |
| **miR-149** | VEGF | CXCR4 | Angiogenesis | Hemangiosarcoma | 127) |
| **miR-181a** | ATM | Stress-sensor kinase |  | Mammary carcinoma | (58) |
| **miR-181d** | P57/Kip2 |  |  | Mammary carcinoma | ((7, 35, 41) |
| **miR-185** | CoLA1 |  | Tumor suppressor | Obstructive sleep apnea canine model | (157) |
| **miR-188** | PR |  |  | Mammary carcinoma | (35) |
| **miR-190a** | NRG3 | ERBB4/PI3k/AKT and MAPK signaling |  | Brain tumor | (100) |
| **miR-194** | CCNE1 | Cyclin |  | Mammary carcinoma | (58) |
| **miR-196a** | ANXA1 | apoptosis |  | Mammary carcinoma | (58) |
| **miR-198** | HER2/ERBB2 |  |  | Mammary carcinoma | (7, 35, 41) |
| **miR-199** |  | Inhibition of ZEB1 and SIP1 expression | Regulates EMT |  | (24) |
| **miR-202** | VEGFA |  | Angiogenesis | Hemangiosarcoma | (127) |
| **miR-203** | ESR1 |  |  | Mammary carcinoma | (7, 35, 41) |
|  | Creb1 | Creb/Mitf/Rab27a pathway | Anti-oncomir, Upregulation of CREB, negative control of the Creb/Mitf/Rab27a pathway | Melanoma | (99, 131) |
| **miR-204** | KRAS | KRAS activated MAPK pathway | Anti-oncomir | Malignant transformation of prostatic hyperplasia | (162) |
| **miR-205** | ERBB3 |  |  | Oral melanoma | (65, 67, 101) |
|  | EGFR |  |  | Mammary carcinoma | (35, 41) |
|  | ECFC | NOTCH2 | Angiogenesis | Distraction osteogenesis | (61) |
| **miR-206** | GCH1 | Inhibition of GCH1 expression, leading to decreased expression of BH4 and NO |  | Atrial fibrillation | (62) |
|  | SOD |  | Regulates ROS | Atrial fibrillation | (63) |
|  | P21/Cip1 |  |  | Mammary carcinoma | (35) |
| **miR-212** | HER2/ERBB2 |  |  | Mammary Carcinoma | (35, 41) |
| **miR-214** | COP1 | P53 pathway for apoptosis | Apoptosis | hemangiosarcoma | (85) |
| **miR-223** | ACVR2A |  |  | Oral melanoma | (65) |
| **miR-299** | P16/INK4A, p14ARF |  |  | Mammary Carcinoma | (35) |
| **miR-300** | NDGR2 |  |  | Oral melanoma | (65) |
| **miR-302d** | TRPC5, NFATC3 |  |  | Mammary carcinoma | (58) |
| **miR-328** | CACNA1C, CACNB1 |  |  | Atrial fibrillation | (56) |
| **miR-374a** | WIF1, PTEN, WNT5A | Negative regulator of the Wnt/β-catenin signaling cascade |  | Mammary Carcinoma | (41, 58) |
| **miR-374b** | PTEN |  |  | Oral Melanoma | (101) |
| **miR-375** | HER2/ERBB2 |  |  | Mammary carcinoma | (35) |
| **miR-429** | HER2/ERBB2 |  |  | Mammary carcinoma | (35) |
| **miR-449** | Pax9 |  |  | Oral melanoma | (65) |
| **miR-451** | CDKI 1B |  | Angiogenesis | hemangiosarcoma | (127) |
| **miR-491** | PTEN | Negative regulator of the AKT/PKB signaling pathway |  | Mammary carcinoma | ((35, 41, 58) |
| **miR-495** | P21/Cip1 |  |  | Mammary carcinoma | (35, 41) |
| **miR-497** | IRAK2 | NF-κβ signaling pathway | Apoptosis | Mammary carcinoma | (163) |
| **miR-502** | VEGFA, angiopoietin 2, PI3K pathway | Inhibits PI3K p85 | Angiogenesis | Hemangiosarcoma | (127) |
| **miR-505** | P16/INK4A, p14ARF |  |  | Mammary Carcinoma | (35) |
| **miR-615** | P16/INK4A, p14ARF |  |  | Mammary Carcinoma | (35) |
| **miR-1306** | JAK-STAT signaling pathway |  |  | H5N1, H3N2 | (145) |
| **miR-1343** | JAK-STAT signaling pathway |  |  | H5N1, H3N2 | (145) |
| **miR-4742** |  | NK cell mediated cytotoxicity, Notch signaling pathway |  | H5N1, H3N2 | (145) |
| **miR-6721** |  | NK cell mediated cytotoxicity, Notch signaling pathways |  | H5N1, H3N2 | (145) |
| **miR-6902** |  | NK cell mediate cytotoxicity, Notch signaling pathway |  | H5N1, H3N2 | (145) |

Abbreviations: ATM: Ataxia Telangiectasia Mutated; BUB1: Budding uninhibited by benzimidazoles 1; CCNA2: Cyclin A2 gene; CCNE1: Cyclin E1 gene; COL1a1: Collagen Type I alpha 1 chain; COL3a1: Collagen Type III alpha 1 chain; COL4a2: Collagen Type IV Alpha 2 Chain; CSF: colony stimulating factor; DDK2: Dickkopf-Related protein 2; EGFR: Epidermal growth factor receptor; ERBB: Epidermal growth factor receptor family; ERK: Extracellular-Signal-Regulated Kinase; HER: Human Epidermal Growth Factor Receptor; ITGA5: Integrin subunit alpha 5; KLF: Krüppel-like factor 4; KRAS: Kristen ras oncogene homolog; MAPK: Mitogen-Activated Protein kinase MET: Hepatocyte growth factor receptor; MTDH: metadherin; PLK1: Polo-like kinase 1; PR: ROCK1: Rho Associated Coiled-Coil Containing Protein Kinase 1; SERP1: Secreted Frizzled- related protein 1; SLC7A11: Solute carrier family member 11; SMAD 7: Mothers against decapentaplegic homolog 7; Spry1: Sprouty homolog 2; TGF-β: Tumor growth factor beta; Timp3: Metalloproteinase inhibitor 3; VEGF: Vascular endothelial growth factor
